# Mucosal-associated invariant T cells contribute to suppression of inflammatory myeloid cells in immune-mediated kidney disease

**DOI:** 10.1038/s41467-023-43269-0

**Published:** 2023-11-15

**Authors:** Ann-Christin Gnirck, Marie-Sophie Philipp, Alex Waterhölter, Malte Wunderlich, Nikhat Shaikh, Virginia Adamiak, Lena Henneken, Tobias Kautz, Tingting Xiong, Daniela Klaus, Pascal Tomczyk, Mohamad M. Al-Bahra, Dirk Menche, Mark Walkenhorst, Olivier Lantz, Anne Willing, Manuel A. Friese, Tobias B. Huber, Christian F. Krebs, Ulf Panzer, Christian Kurts, Jan-Eric Turner

**Affiliations:** 1 III. Department of Medicine, University Medical Center Hamburg-Eppendorf, Hamburg, Germany; 2 Hamburg Center for Translational Immunology, University Medical Center Hamburg-Eppendorf, Hamburg, Germany; 3 Hamburg Center for Kidney Health (HCKH), University Medical Center Hamburg-Eppendorf, Hamburg, Germany; 4 Institute of Molecular Medicine and Experimental Immunology, University Hospital Bonn, Bonn, Germany; 5 Kekulé Institute of Organic Chemistry and Biochemistry, University of Bonn, Bonn, Germany; 6 Institute of Neuroimmunology and Multiple Sclerosis, University Medical Center Hamburg-Eppendorf, Hamburg, Germany; 7 Inserm U932, Laboratoire d’immunologie Clinique and Centre d’investigation Clinique en Biothérapie Gustave-Roussy, Institut Curie, Paris, France; 8 Department of Microbiology and Immunology, Doherty Institute for Infection and Immunity, University of Melbourne, Melbourne, VIC Australia; 9 Present Address: Euroimmun, Lübeck, Germany; 10 Present Address: Division of Immunology, Paul-Ehrlich-Institut Langen, Langen, Germany; 11 Present Address: Institut für Transfusionsmedizin, University Medical Center Hamburg-Eppendorf, Hamburg, Germany

**Keywords:** Nephrology, Nephritis, Innate lymphoid cells, RNA sequencing

## Abstract

Mucosal-associated invariant T (MAIT) cells have been implicated in various inflammatory diseases of barrier organs, but so far, their role in kidney disease is unclear. Here we report that MAIT cells that recognize their prototypical ligand, the vitamin B2 intermediate 5-OP-RU presented by MR1, reside in human and mouse kidneys. Single cell RNAseq analysis reveals several intrarenal MAIT subsets, and one, carrying the genetic fingerprint of tissue-resident MAIT17 cells, is activated and expanded in a murine model of crescentic glomerulonephritis (cGN). An equivalent subset is also present in kidney biopsies of patients with anti-neutrophil cytoplasmatic antibody (ANCA)-associated cGN. MAIT cell-deficient MR1 mice show aggravated disease, whereas B6-MAIT^CAST^ mice, harboring higher MAIT cell numbers, are protected from cGN. The expanded MAIT17 cells express anti-inflammatory mediators known to suppress cGN, such as CTLA-4, PD-1, and TGF-β. Interactome analysis predicts CXCR6 – CXCL16-mediated cross-talk with renal mononuclear phagocytes, known to drive cGN progression. In line, we find that cGN is aggravated upon CXCL16 blockade. Finally, we present an optimized 5-OP-RU synthesis method which we apply to attenuating cGN in mice. In summary, we propose that CXCR6^+^ MAIT cells might play a protective role in cGN, implicating them as a potential target for anti-inflammatory therapies.

## Abstract (web summary)

Mucosal associated invariant T (MAIT) cells reside in barrier organs, but their contribution to inflammatory processes in the kidneys is not fully known. Here authors find by single cell RNA sequencing that among the different MAIT cell subtypes found at steady state, a population with MAIT17 signature is expanded in both human crescentic glomerulonephritis and its mouse model, and these cells may play protective role in the disease.

## Introduction

Pathogenic immune responses in the kidney often commence in the glomerulus, leading to glomerulonephritis that may spread over the tubulointerstitium and cause progressive impairment of kidney function, ultimately resulting in end-stage kidney disease. As a disease group, the different glomerulonephritis forms underlie up to 20% of chronic kidney diseases in industrialized countries and hence represent a considerable financial burden for healthcare systems^1^. The most aggressive form of glomerulonephritis (GN) is characterized by proliferation of Bowman’s capsule parietal epithelial cells which, together with immune cell infiltration around and within the glomeruli, results in characteristic crescents. In most cases, crescentic GN (cGN) patients require intense immunosuppression to stop or delay disease progression, but the therapeutic regimens available are unspecific, hampered by limited effectiveness, and often show severe systemic side-effects^1,2^.

In recent years, numerous studies have elucidated the mechanisms underlying cGN by using the nephrotoxic nephritis model that mimics the histopathology and T cell-mediated immune responses of human cGN^2–4^. The early inflammatory phase is executed by cells of the innate immune system, followed by a pathogenic intrarenal T_H_17 response, and, at later stages, by an injurious T_H_1-mediated delayed type hypersensitivity reaction regulated by dendritic cells. Also anti-inflammatory players that partially downregulate the pathogenic immune response in experimental cGN have been identified, such as NKT cells in the T_H_17 phase and regulatory T cells in the T_H_1 phase^2,4^.

Recent immunological studies have highlighted the role of T cells with invariant T cell receptors (TCR) in various inflammatory and autoimmune diseases, such as Mucosal-associated invariant T (MAIT) cells, which express a semi-invariant T cell receptor, consisting of TRAV1-2 joined to TRAJ33 in mice and humans^5^. These cells are restricted by the non-polymorphic MHC class I-like protein MR1 and recognize vitamin B2 metabolites produced by certain bacteria^6,7^. In most murine tissues, MAIT cells display a T-bet^+^ IFN-γ-producing MAIT1 or RORγt^+^ IL-17A-producing MAIT17 phenotype. Upon antigen recognition of bacterial metabolites, MAIT cells are activated, secrete various cytokines, and perform cytotoxic functions by producing granzyme B and perforin^8–13^. In addition to TCR-mediated antigen recognition, MAIT cells can be directly activated by cytokines, mainly IL-12 and IL-18, that are produced during viral infections^14,15^. These differential activation modes enable them to respond to bacterial and viral infections. While such antimicrobial functions of MAIT cells have been well documented, their role in non-infectious immune-mediated inflammatory diseases (IMID), especially glomerulonephritis, remains unclear^16,17^.

So far, most studies on MAIT cells in IMID patients, including anti-neutrophil cytoplasmatic antibody (ANCA)-associated vasculitis with cGN^18,19^, are limited to analyses of peripheral blood MAIT cells. These studies showed an activated, proinflammatory and/or exhausted phenotype of MAIT cells and reduced peripheral frequencies, suggesting their recruitment to inflammatory sites^16,17^. In contrast, several recent transcriptomic analyses of human and murine MAIT cells described upregulation of a tissue repair signature after activation, indicating that these cells might also play a role in tissue repair after injury^20–22^.

Although initially identified in mucosal tissues, MAIT cells also populate internal organs. Indeed, limited evidence from experimental models of sterile inflammation of the central nervous system^23,24^, the pancreas^25^, and the liver^26^ supports a tissue protective potential of MAIT cells in certain experimental settings. Recent reports have identified MAIT cells also in kidneys of humans and mice^27–29^. Some observations in a lupus nephritis model hinted at proinflammatory functions^30^, but the detailed role of MAIT cells in kidney inflammation is largely unclear.

Here, we present the first evidence for a protective role of MAIT cells in renal inflammation. Single-cell RNA sequencing (scRNAseq) analysis of MAIT cells residing in the mouse and human kidney reveals a MAIT17 subset that expands and gets activated during renal inflammation. These MAIT17 cells express anti-inflammatory mediators and interact with myeloid cells in the kidney tissue which might result in suppression of proinflammatory functions of the latter and thereby ameliorate cGN. In particular, we report that pharmacological MAIT cell activation attenuates a model of cGN, which might help to develop MAIT-cell-based therapeutic interventions in the future.

## Results

### MAIT17 cells reside in the human kidney and are activated in ANCA-associated glomerulonephritis

**Fig. 1:**
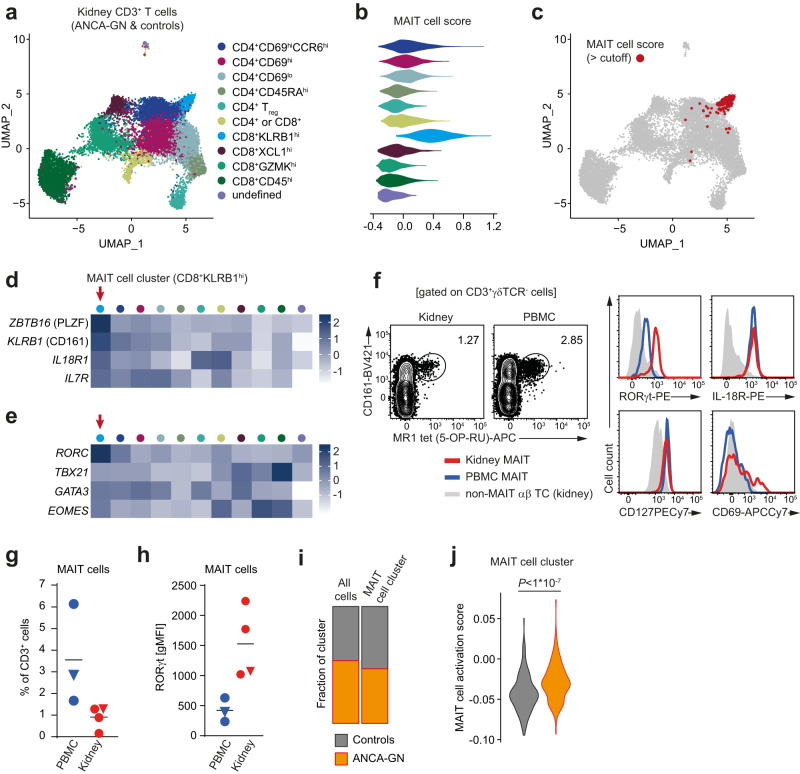
Mucosal-associated invariant T (MAIT) 17 cells reside in the human kidney and are activated in anti-neutrophil cytoplasmatic antibody (ANCA)-associated glomerulonephritis (GN). **a** Unsupervised Uniform Manifold Approximation and Projection (UMAP) clustering (resolution 0.6) of a published data set^31^ of pooled single-cell RNA sequencing (scRNAseq) data from CD3^+^ T cells isolated from kidney biopsy specimens of ANCA-GN patients (*n* = 6) and unaffected kidney tissue of tumor nephrectomy patients (controls, *n* = 3). Eleven clusters were identified and named according to cluster defining genes. **b** Expression of an uncurated MAIT cell-defining gene signature generated from a MAIT cell cluster identified in the bronchoalveolar lavage fluid of Coronavirus Disease 2019 patients^32^ (MAIT cell score, see Supplementary Fig. 1) in the indicated clusters. Color coding as in (**a**). **c** Cells that show high expression of the MAIT cell score were marked in red and plotted on the original UMAP plot. Gene expression heat map of MAIT cell-defining genes (**d**) and T cell lineage-defining transcription factors (**e**) in the indicated clusters. Color coding as in (**a**). **f** Representative flow cytometric identification and characterization of MAIT cells in paired kidney and blood samples of a tumor nephrectomy patient. Gating strategy is specified in brackets and numbers indicate the percentage of cells in the gate. MAIT cell frequencies in the CD3^+^ T cell fraction (**g**) and RORγt expression in MAIT cells (**h**) from unaffected kidney tissue and blood of tumor nephrectomy patients, as well as from the blood of healthy human donors. Samples from the same patient are marked with a triangle symbol. Each symbol in (**g**) and (**h**) represents one biologically independent sample (*n* = 3 for PBMC, *n* = 4 for kidney) and horizontal lines represent the mean. **i** Contribution of cells from controls and ANCA-GN patients to the MAIT cell cluster (CD8^+^KLRB1^hi^) identified in scRNAseq analyses. **j** Expression of an uncurated gene signature defining in vitro activated human peripheral blood MAIT cells (MAIT cell activation score (Hinks et al.^22^)) in the MAIT cell cluster of controls and ANCA-GN patients. The two-sided Student’s *t* test was used for comparison between the two groups. (UMAP uniform manifold approximation and projection for dimension reduction, ANCA-GN anti-neutrophil cytoplasmic antibody-associated glomerulonephritis, PBMC peripheral blood mononuclear cells, gMFI geometric mean fluorescent intensity). Source data are provided as a Source Data file.

Recent reports indicate the presence of MAIT cells in human kidney tissue^27–29^, but their expression profile and potential contribution to renal inflammation has not been addressed so far. For a detailed profiling of human kidney MAIT cells in homeostasis and renal autoimmunity, we first re-analyzed a scRNAseq dataset of kidney T cells that we recently published^31^. A total of 12,734T cells from controls (*n* = 3) and patients with ANCA-associated glomerulonephritis (ANCA-GN) (*n* = 6), a severe form of renal autoimmunity with cGN, were combined and subjected to unsupervised clustering by the UMAP algorithm (Fig. 1a). As previously described^31^, we identified 11 subclusters of CD4^+^ and CD8^+^ T cells with specific mRNA expression profiles (Fig. 1a) that were labeled according to cluster-defining genes (Supplementary Fig. 1A). Due to the limited sequencing depth we were unable to identify MAIT cells in this data set by their specific T cell receptor (TCR) sequences (e.g., *TRAV1-2*). Therefore, we aimed at identifying kidney MAIT cells in the ANCA-GN dataset by a broader gene expression profile specific for MAIT cells. To this end, we extracted a MAIT cell-defining gene expression module from an unrelated scRNAseq dataset of T cells isolated from the bronchoalveolar lavage fluid (BALF) of Coronavirus Disease 2019 (COVID19) patients, recently published by our group^32^. This more recent data set with improved sequencing depth allowed for unequivocal identification of TCR Vα7.2^+^ *TRAV1-2*-expressing MAIT cells by epitope measurement using barcoded antibodies (cellular indexing of transcriptomes and epitopes by sequencing = CITE-seq) (Supplementary Fig. 1B). Of note, the markers used to identify BALF MAIT cells by CITE-seq in this data set were also expressed on kidney MAIT cells, as demonstrated by flow cytometry (Supplementary Fig. 1C). The uncurated MAIT cell gene expression module (MAIT cell score) used to identify kidney MAIT cells in the ANCA-GN data set was composed of the 20 cluster-defining genes of the MAIT cell cluster in the BALF COVID19 data set (Supplementary Fig. 1D). The quantification of MAIT cell score expression in the 11 renal T cell clusters defined in Fig. 1a revealed that the *CD8*^+^*KLRB1*^hi^ (the latter encoding for CD161) cluster showed an mRNA expression profile consistent with MAIT cells (Fig. 1b, d). Moreover, overlaying cells that showed high expression of the MAIT cell score onto the original UMAP plot confirmed that the vast majority of these cells were located in this *CD8*^+^*KLRB1*^hi^ cluster (Fig. 1c). Since NKT cells might show a similar gene expression profile, we wished to exclude contamination of the identified MAIT cell cluster with this other innate-like T cell population. By using a similar strategy as described above for the MAIT cell score, we extracted a NKT cell-defining expression score (top 20 cluster defining genes of the NKT cell cluster) from the BALF COVID19 data set (Supplementary Fig. 2A–C) and overlaid cells with high NKT cell score expression onto the ANCA-GN data set (Supplementary Fig. 2D–G). These analyses confirmed that NKT cells did not contribute to the MAIT cell cluster.

To determine the predominant MAIT cell subset in the kidney, we analyzed the expression of T cell lineage-defining transcription factors in the 11 clusters that revealed high expression of *RORC* (encoding for the retinoic acid receptor-related orphan receptor C, RORγt) in the MAIT cell population, identifying them as MAIT17 cells (Fig. 1e and Supplementary Fig. 1D). Next, we performed specific MAIT cell flow cytometric analyses with 5-OP-RU loaded MR1-tetramer (MR1 tet(5-OP-RU)) in control kidneys and blood samples, confirming high protein expression of RORγt in renal MAIT cells compared to their circulating counterparts (Supplementary Fig. 1E and Fig. 1f–h). The comparison of kidney MAIT cell frequency between controls and patients with ANCA-GN in the scRNAseq dataset showed that the proportion of MAIT cells in the total renal T cell population was unchanged in ANCA-GN (Fig. 1i). However, application of a MAIT cell activation score, composed of a module of genes upregulated in human peripheral blood MAIT cells after in vitro activation with 5-OP-RU-loaded MR1 tetramers^22^, to the cell clusters showed significant MAIT cell activation in ANCA-GN (Fig. 1j). In summary, these data indicate the presence of MAIT17 cells in healthy and inflamed human kidney tissue and are consistent with their activation in autoimmune renal inflammation in ANCA-associated small vessel vasculitis.

### MAIT cells display a tissue-resident MAIT17 phenotype in the murine kidney

**Fig. 2:**
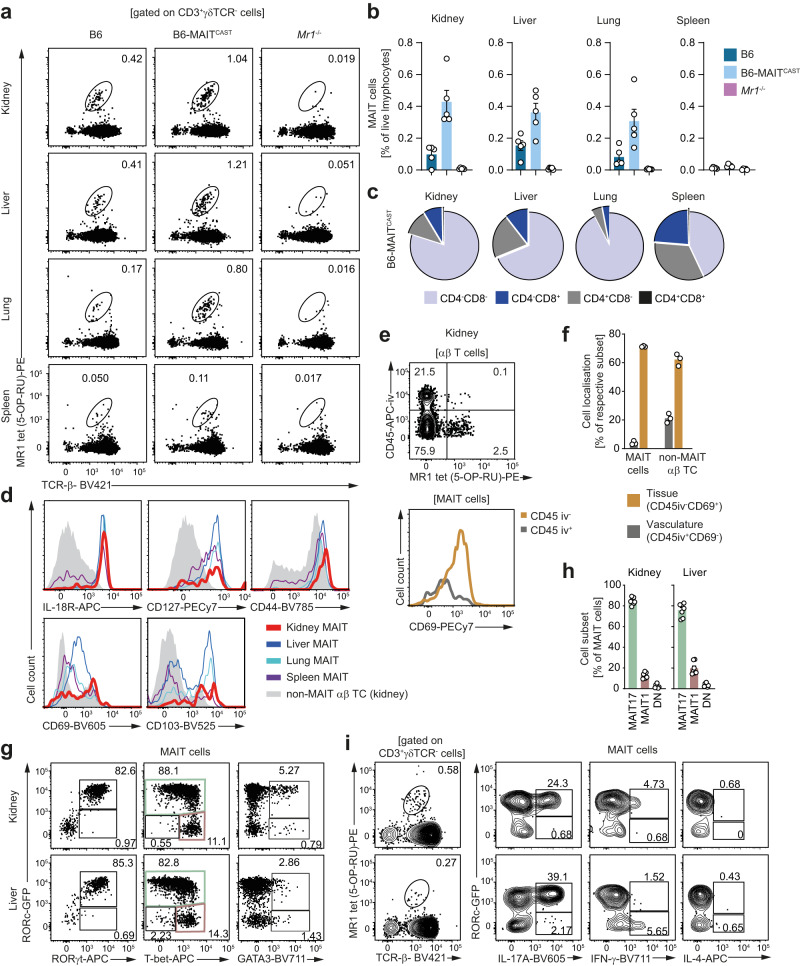
Mucosal-associated invariant T (MAIT) cells display a tissue-resident MAIT17 phenotype in the murine kidney. **a** Representative flow cytometry plots of leukocytes isolated from kidney, liver, lung, and spleen of naïve B6, B6-MAIT^CAST^, and *Mr1*^*−/−*^ mice. **b** Quantification of MAIT cell frequencies in the live lymphocyte gate in the respective organs and mouse strains (see (**a**)). **c** Distribution of CD4 and CD8 surface expression in the MAIT cell population of naïve B6-MAIT^CAST^ mice in kidney, liver, lung, and spleen. **d** Representative flow cytometric analysis of surface marker expression on MAIT cells in kidney, liver, lung, and spleen of naïve B6-MAIT^CAST^ mice. **e** Representative flow cytometry plots of αβ T cells from kidneys of naïve B6-MAIT^CAST^ mice euthanized shortly after intravenous (i.v.) injection of an fluorochrome coupled α-CD45 antibody (upper panel) and histogram of CD69 surface expression in the CD45 iv^+^ and CD45 iv^−^ MAIT cell population (lower panel). **f** Distribution of CD45 iv^+^ CD69^−^ (intravascular) and CD45 iv^−^ CD69^+^ (tissue) populations in the MAIT cell and non-MAIT αβ T cell compartment in the kidneys of naïve B6-MAIT^CAST^ mice (*n* = 3 biologically independent animals per group). Representative flow cytometry plots of transcription factor expression (**g**) and quantification of MAIT cell subsets (RORc-GFP^+^T-bet^+/−^ = MAIT17, RORc-GFP^-^T-bet^+^ = MAIT1, RORc-GFP^−^T-bet^−^ = double negative (DN)) (**h**) in kidney and liver MAIT cells from naïve RORc-GFP B6-MAIT^CAST^ mice (*n* = 7 biologically independent animals per group). **i** Cytokine production (after restimulation with phorbol 12-myristate 13-acetate/ionomycin) in kidney and liver MAIT cells from naïve RORc-GFP B6-MAIT^CAST^ mice. Gating strategies are specified in brackets and numbers indicate percentage of cells in the gates. Bars represent mean ± SEM and symbols represent individual animals. All experiments were performed twice with at least three mice per group. Source data are provided as a Source Data file.

In order to investigate the potential functional significance of a kidney-residing MAIT17 cell population in a preclinical model, we addressed the question whether the kidney of laboratory mice harbors a comparable population of MAIT cells. The gating strategy for analysis of MAIT cells in mice is depicted in Supplementary Fig. 3A. Since tissue-residing and circulating MAIT cell numbers in the standard C57BL/6 (B6) mouse strains are relatively low compared to those in humans, we additionally employed the widely used congenic B6-MAIT^CAST^ mouse strain that contains a genetic trait of wild-derived inbred CAST/EiJ mice, leading to a substantial increase in MAIT cell abundance^33^. MAIT cell-deficient *Mr1*^−/−^ mice were used as negative controls^6^. Indeed, specific flow cytometric analyses of MAIT cells from kidney and various other organs with MR1-tet(5-OP-RU) showed comparable frequencies of MAIT cells in the kidney compared to the liver and lung, organs in which these cells have been shown to play important roles in the immune response (Fig. 2a, b and Supplementary Fig. 3D)^16^. Moreover, MAIT cell frequencies in the kidney of naïve B6-MAIT^CAST^ mice were similar to those in healthy human kidney tissue (see Figs. 1g and 2a). Consistent with the MAIT cell phenotype in other anatomical locations, detailed flow cytometric characterization of kidney MAIT cells in B6-MAIT^CAST^ mice revealed a predominantly CD4^−^CD8^−^ phenotype, high expression of IL-18R, CD127, and CD44, as well as of the tissue residency markers CD69 and CD103 (Fig. 2d). Moreover, renal MAIT cells were almost exclusively negative for staining with an intravenously injected CD45 antibody, confirming their location within the kidney tissue (Fig. 2e, f). An assessment of transcription factor expression profiles revealed that the vast majority of kidney MAIT cells in B6-MAIT^CAST^ and C57BL/6 mice were positive for RORγt, intermediate for T-bet, and negative for GATA-3 (Fig. 2g, h and Supplementary Fig. 3E). Consistent with the RORγt^+^ MAIT17 profile, IL-17A was the main lineage-defining cytokine produced by MAIT cells isolated from the kidney and restimulated with PMA and ionomycin (Fig. 2i). Taken together, these analyses show that murine kidney MAIT cells show a tissue-resident MAIT17 phenotype that resembles basic characteristics of MAIT cells in human kidneys.

### Kidney MAIT17 cells express pro- and anti-inflammatory mediators in experimental cGN

**Fig. 3:**
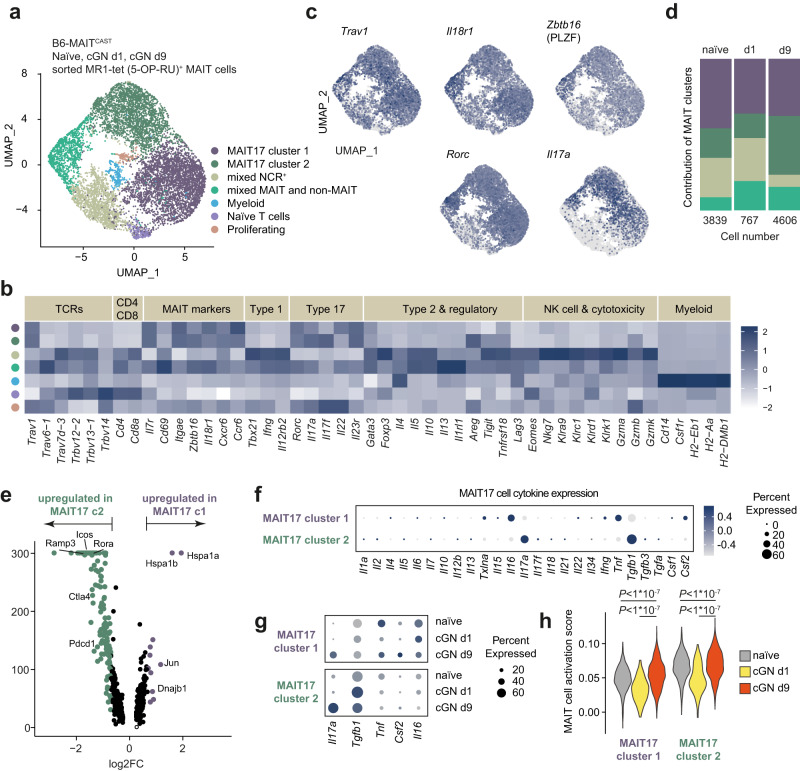
Kidney mucosal-associated invariant T (MAIT) 17 cells express pro- and anti-inflammatory mediators in experimental crescentic glomerulonephritis (cGN). **a** Uniform Manifold Approximation and Projection (UMAP) dimensionality reduction (resolution 0.2) of pooled single-cell RNA sequencing data of MR1 tetramer (5-OP-RU)^+^ MAIT cells isolated by flow cytometric cell sorting from kidneys of naïve B6-MAIT^CAST^ mice and on day 1 and 9 after induction of cGN (naïve *n* = 18, cGN day 1 *n* = 11, cGN day 9 *n* = 13; *n* represents the number of biologically independent, male, 9–14 week-old mice that were pooled for the isolation). Seven clusters were identified and named according to cluster defining genes and expression of selected marker genes. **b** Heat map of normalized marker gene expression of the indicated clusters. **c** UMAP plots showing expression of MAIT cell-defining genes (upper panels) and MAIT17 cell markers in the identified clusters. **d** Contribution of different MAIT cell clusters to the dataset. Numbers below the bars indicate total number of MAIT cells analyzed for each time point. **e** Volcano plot of differential gene expression in MAIT17 clusters 1 and 2. **f** Gene expression of all interleukins expressed in the data set and other selected cytokines in MAIT17 cluster 1 and 2. **g** Expression of selected cytokine genes in MAIT17 clusters 1 and 2 at the different time points. **h** Expression of an uncurated gene signature defining in vivo activated murine MAIT cells (MAIT cell activation score (Hinks et al.^22^)) in MAIT cell clusters 1 and 2 at the different time points. One-way ANOVA was used for statistical analysis followed by a post hoc analysis with Newman-Keuls test for multiple comparisons.

After establishing the B6-MAIT^CAST^ mouse strain as a suitable model for human kidney MAIT cell biology, we induced a widely used experimental model for cGN (nephrotoxic serum nephritis)^3,34^ that mimics features of human ANCA-GN in these mice. To provide a deep profiling of MAIT cells at different time points of kidney inflammation, we isolated MR1-tet(5-OP-RU)^+^ MAIT cells from the kidneys of naïve B6-MAIT^CAST^ mice and on days 1 and 9 after induction of cGN and subjected them to scRNAseq. The sorting strategy for isolation of MAIT cells is depicted in Supplementary Fig. 3B. These analyses yielded a total of 9212 cells that divided into seven subsets by unsupervised UMAP clustering, two of which were composed of MAIT17 cells, as indicated by high expression of MAIT cell-defining genes such as the *Trav1* (coding for the MAIT cell TCR), *Il18r1*, *Zbtb16* (encoding for PLZF), *Rorc*, *Il17a*, and *Il23r* (Fig. 3a–c and Supplementary Fig. 4). From the remaining minority of cells, one smaller cluster expressed *Trav1* along with other non-MAIT TCR genes, *Cd4*, *Cd8*, as well as a mixture of type 1, type 2, and type 17 markers, indicating contamination with non-MAIT CD4^+^ and CD8^+^ T cells. Another *Trav1*-low cluster showed high expression of NK cell and type 1 markers, as well as cytotoxic molecules, indicating NKT cell origin.

The two major clusters of MAIT17 cells comprised 50–80% of cells depending on the time point. Interestingly, both MAIT17 cell cluster 1 (c1) and MAIT17 cell cluster 2 (c2) were present in the kidney of naïve mice, but only c2 expanded with the progression of cGN (naïve and day 1 vs. day 9), whereas c1 remained unchanged in frequency (Fig. 3d and Supplementary Fig. 4A). Unbiased differential expression analysis of MAIT17 c1 vs. c2 showed an upregulation of several cell contact-dependent stimulatory and inhibitory immune cell regulators such as *Icos*, *Ctla4*, and *Pdcd1* (encoding for PD-1) in MAIT17 c2 cells (Fig. 3e). To explore whether MAIT17 cells might employ additional cytokines, apart from IL-17A, to regulate the immune response in the kidney, we analyzed mRNA expression of all known interleukins and a selected list of other cytokines in the two clusters predominant in murine kidney inflammation (Fig. 3f, g). Unexpectedly, in addition to IL-17A, these analyses revealed a strong expression of *Tgfb1* (encoding for TGF-β) in MAIT17 c2 cells, a cytokine that has been implicated in the downregulation of inflammation in cGN^35^. While IL-17A production was low in both MAIT17 cell clusters from naïve mice, it appeared to be upregulated with the progression of cGN in both clusters (day 1 vs. day 9). In contrast, TGF-β seemed to be a feature of kidney MAIT c2 cells already in homeostatic conditions and at an early disease stage (day 1) (Fig. 1g). In analogy to the analyses of MAIT cell activation in human control vs. ANCA-GN kidney tissue (see Fig. 1j), we extracted a gene list for a mouse MAIT cell activation score from a recent publication providing bulk RNAseq data of resting vs. activated mouse MAIT cells^22^. The application of this gene module to the MAIT17 clusters clearly indicated an increase in MAIT cell activation at the late time point of cGN (day 9 vs. day 1 and naïve) and an overall higher activation score in cluster c2 (Fig. 3h).

In summary, these scRNAseq analyses identify a MAIT17 cell cluster in the murine kidney, present in homeostasis, that expands and shows increased activation with the progression of cGN, expressing proinflammatory (IL-17A and ICOS) as well as anti-inflammatory (CTLA4, PD-1, and TGF-β) mediators that might regulate the renal immune response.

### MAIT cells protect from renal tissue damage in experimental cGN

**Fig. 4:**
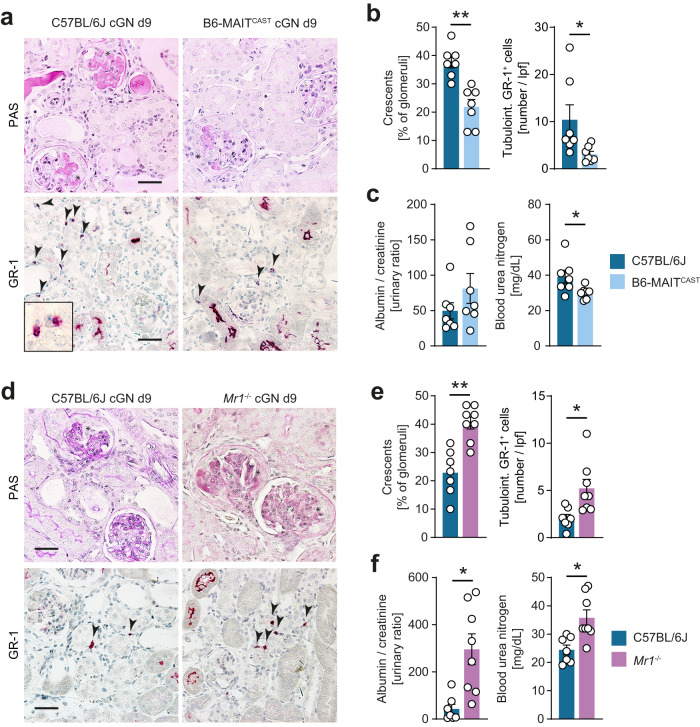
Mucosal-associated invariant T (MAIT) cells protect from renal tissue damage in experimental crescentic glomerulonephritis (cGN). **a** Representative photomicrographs of kidney sections from C57BL/6J wild type and B6-MAIT^CAST^ mice on day 9 of cGN stained with periodic acid-Schiff (PAS) (upper panel) and immunohistochemistry for the neutrophil marker GR-1 (lower panel). Asterisks mark glomerular crescents, arrow heads mark neutrophils in the tubulointerstitial area. **b** Quantification of crescent formation and tubulointerstitial neutrophil infiltration (C57BL/6J wild type *n* = 7, B6-MAIT^CAST^ *n* = 7; *n* represents the number of biologically independent animals). **c** Kidney function parameters in C57BL/6J wild type and B6-MAIT^CAST^ mice (groups as in **b**). Data in (**a**–**c**) are representative for three independent experiments. **d** Representative PAS and GR-1 stainings of kidney sections from C57BL/6J wild type and *Mr1*^*−/−*^ mice on day 9 of cGN. Asterisks mark glomerular crescents, arrow heads mark neutrophils in the tubulointerstitial area. **e** Quantification of crescent formation and tubulointerstitial neutrophil infiltration (C57BL/6J wild type *n* = 7, *Mr1*^*−/−*^ *n* = 8; *n* represents the number of biologically independent animals). **f** Kidney function parameters in C57BL/6J wild type and *Mr1*^*−/−*^ mice (C57BL/6J wild type *n* = 7, *Mr1*^*−/−*^ *n* = 8; *n* represents the number of biologically independent animals). Data in (**d**–**f**) are representative for two independent experiments. Scale bar represents 20 µm. Symbols represent individual animals. Bars represent mean ± SEM. (*p < 0.05, **p < 0.01 in two-sided Student’s *t* test). Source data are provided as a Source Data file.

To functionally validate the effect of MAIT cells in renal inflammation, we performed gain- and loss-of-function experiments, comparing the histopathological and clinical outcomes of B6 (low MAIT cell frequency), B6-MAIT^CAST^ (4-fold increase in MAIT cell frequency, see Fig. 2a, b), and *Mr1*^−/−^ (MAIT cell deficiency) mice in experimental cGN. In severe cGN, increased MAIT cell frequency in B6-MAIT^CAST^ mice (as compared to B6) correlated with significant renal tissue protection with reduced crescent formation, and tubulointerstitial inflammation as indicated by reduced GR-1^+^ neutrophil infiltration into the kidney (Fig. 4a, b). While albuminuria was unchanged, we also observed a significant improvement in kidney function in B6-MAIT^CAST^ mice (reduced blood urea nitrogen levels) compared with that in B6 controls (Fig. 4c). As shown in Fig. 2, renal MAIT cell numbers were low in naïve B6 mice but we could demonstrate a 4–5-fold inflammation-induced increase at day 9 of cGN (Supplementary Fig. 5). In line with the hypothesis that MAIT cells have regulatory functions, the comparison of nephritic B6 mice with *Mr1*^−/−^ mice in cGN of moderate severity revealed significant aggravation of histopathological and clinical parameters (Fig. 4d–f), indicating that even the low numbers of MAIT cells in B6 mice might limit renal tissue damage.

### Blocking MR1-dependent MAIT cell activation does not influence the course of experimental cGN

To determine whether the protective MAIT cell activation requires TCR–MR1 interaction, we performed MR-1 blockade experiments by applying an α-MR1 antibody^36^ to C57BL/6 (Supplementary Fig. 6) and B6-MAIT^CAST^ mice (Supplementary Fig. 7) with cGN. The application of this antibody did not significantly alter the histopathological or clinical outcome of cGN in either strain, as measured by crescent formation, tubulointerstitial inflammation, blood urea nitrogen levels, and albuminuria. These data suggest that in the cGN model, in which microbe-derived MAIT-TCR ligands are presumably absent, other TCR-independent pathways for MAIT cell activation, such as stimulation via cytokine receptors, might be predominant.

### Immunomodulatory MAIT17 cells interact with proinflammatory mononuclear phagocytes in experimental cGN

**Fig. 5:**
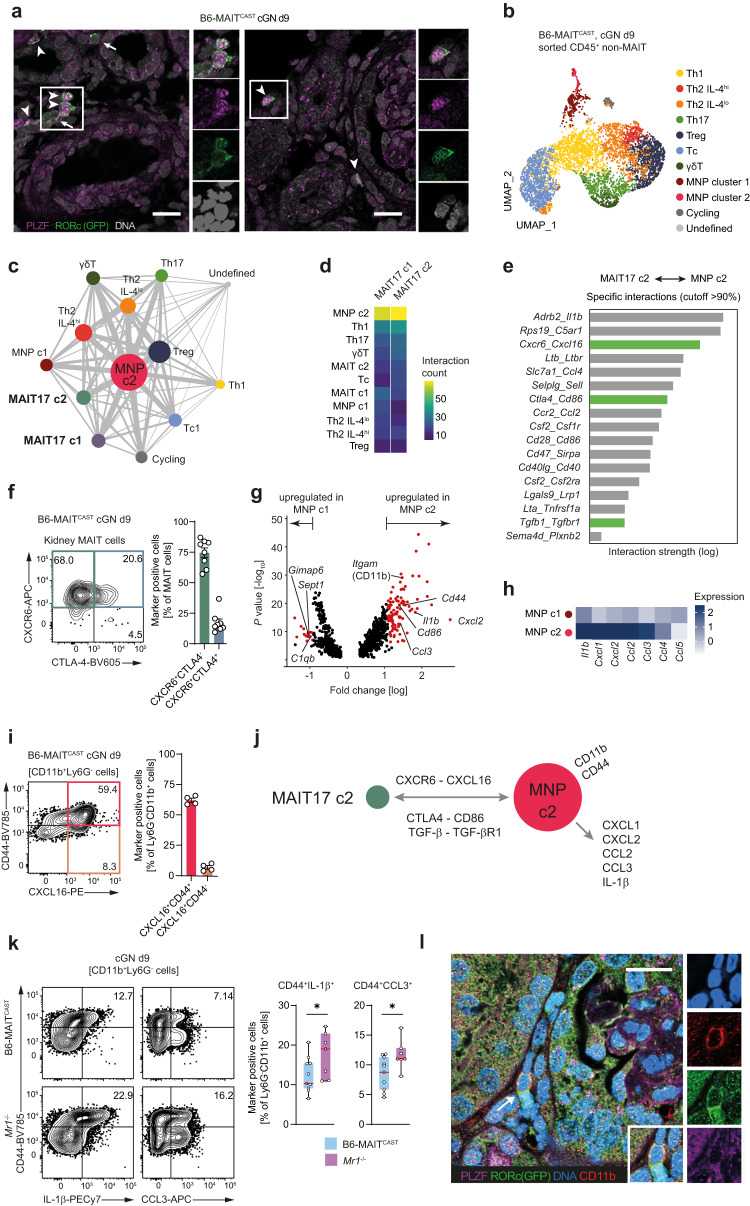
Immunomodulatory mucosal-associated invariant T (MAIT) 17 cells interact with proinflammatory monocytes in experimental crescentic glomerulonephritis (cGN). **a** Representative immunofluorescence staining for identification of PLZF^+^RORc^+^ MAIT17 cells in kidney sections of RORc-GFP B6-MAIT^CAST^ mice on day 9 after cGN induction (representative for *n* = 3 biologically independent animals). **b** Uniform Manifold Approximation and Projection (UMAP) dimensionality reduction (resolution 0.5) of single-cell RNA sequencing data of CD45^+^ non-MAIT cells isolated by flow cytometric cell sorting from kidneys of B6-MAITCAST mice on 9 after induction of cGN. The 11 clusters were named according to cluster defining genes and expression of selected marker genes. **c** Interaction network based on ligand–receptor interactions between all CD45^+^ cell clusters identified in kidneys of B6-MAIT^CAST^ mice with cGN (pooled non-MAIT cell and MAIT cell data sets). Dot size represents the number of total significant interactions with other clusters and line thickness represents number of interactions between individual clusters. **d** Heatmap showing the absolute number of significant interactions between the indicated cell clusters. **e** List of top ligand–receptor interactions between MAIT17 cluster 2 and mononuclear phagocyte (MNP) cluster 2 filtered for interaction strength (>1) and specificity (>90%). Interaction pathways that have been implicated in amelioration of cGN are marked in green. **f** Representative flow cytometry plot and quantification of CXCR6 and CTLA4 expression in kidney MAIT cells of B6-MAIT^CAST^ (cGN day 9, *n* = 8 biologically independent animals). **g** Volcano plot of differential gene expression between MNP clusters 1 and 2. Significantly upregulated genes are marked in red. **h** Normalized gene expression heatmap of proinflammatory cytokines and chemokines MNP clusters 1 and 2. **i** Representative flow cytometry plot and quantification of CXCL16 and CD44 expression (after restimulation with PMA/ionomycin) in kidney CD11b^+^ MNPs from B6-MAIT^CAST^ mice (cGN day 9, *n* = 4 biologically independent animals). **j** Hypothetic working model for interaction between MAIT17 cluster 2 and MNP cluster 2 based on the interactome analysis. **k** Representative flow cytometry plots and quantification of IL-1β and CCL3 production (after restimulation with phorbol 12-myristate 13-acetate/ionomycin) by CD11b^+^CD44^hi^ MNPs in the kidney of B6-MAIT^CAST^ and *Mr1*^*−/−*^ mice with cGN (day 9, B6-MAIT^CAST^ *n* = 11 biologically independent animals, *Mr1*^*−/−*^ *n* = 7 biologically independent animals). **l** Representative immunofluorescence staining of MAIT17 cells located in close proximity to CD11b^+^ mononuclear phagocytes (arrow) in kidney sections of B6-MAIT^CAST^ mice (cGN day 9, representative for *n* = 3 biologically independent animals). Data in (**f**) and (**k**) are pooled from two independent experiments. Data in (**a**, **l**) are representative for two independent experiments with *n* = 3 biologically independent animals with similar results. Scale bar represents 15 µm. Circles in bar graphs represent individual animals and bars represent mean ± SEM. (*p < 0.05 in two-sided Student’s *t* test). Source data are provided as a Source Data file.

To understand the mechanisms that govern MAIT cell responses within the kidney, we studied their intrarenal location in murine cGN in B6-MAIT^CAST^ mice. Using immunofluorescence staining for PLZF and RORc-GFP, we could clearly identify PLZF^+^RORc^+^ MAIT cells in the kidney that were mainly located in the tubulointerstitial and periglomerular infiltrates (Fig. 5a). These infiltrates are a histopathological hallmark of human and experimental cGN^2^.

To identify the immune cell types within these infiltrates that interact with MAIT cells, we isolated CD45^+^ non-MAIT cells from the kidney at day 9 of cGN and performed scRNAseq. Unsupervised UMAP clustering of the data of 5271 cells retrieved from these analyses identified seven T cell subsets and two major myeloid cell subtypes that were labeled according to their expression of cluster-defining genes and selected leukocyte subset markers (Fig. 5b and Supplementary Fig. 8A, B). Based on these clusters and the two predominant MAIT17 cell clusters in nephritic kidneys identified in Fig. 3, we calculated the interactome of all immune cells found in the kidney in experimental cGN using the CellPhone database^37,38^. The network analyses of transcriptome-based ligand–receptor interactions between cell types showed a node of mononuclear phagocytes (MNP) at the center that was characterized by a high number of potential interactions with different T cell subsets (Fig. 5c). Interestingly, the MAIT17 c2 cell cluster, previously identified to express cell contact-dependent anti-inflammatory molecules, demonstrated a high number of interactions with this MNP cluster c2, much more than for example with MNP cluster c1 or other T cell subsets (Fig. 5c, d). We therefore decided to investigate the specific interactions of MAIT17 c2 with MNP c2 more closely. After removing unspecific interactions (cut off 90%), 17 interactions with a strength >1 remained, three of which were well-described immunosuppressive pathways that had previously been reported to suppress inflammation in murine models of cGN (Fig. 5e)^35,39,40^. Indeed, the expression of the top two hits in these analyses, CXCR6 and CTLA-4, on murine MAIT cells could be confirmed on protein basis by flow cytometry (Fig. 5f).

Next, we performed differential expression analysis of MNP c2 vs. MNP c1 which indicated that MNP c2 cells express high levels of *Cd11b*, *Cd44*, and *Cd86* (an interaction partner of CTLA-4), as well as the proinflammatory mediators *Cxcl2*, *Ccl3*, and *Il1b* (Fig. 5g, h). Moreover, flow cytometry confirmed that CD11b^+^CD44^hi^ MNPs expressed high levels of CXCL16 (the ligand for CXCR6) (Fig. 5i). The gating strategy for analysis CD11b^+^Ly6G^−^ MNP in mice is depicted in Supplementary Fig. 3C. These interactome analysis defined a working model, where the CXCR6-CXCL16 axis acts as a co-localization signal of MAIT17 cells toward proinflammatory MNPs in the kidney, resulting in MNP suppression, perhaps through MAIT cell-expressed CTLA-4 and/or TGF-β (Fig. 5j).

In line with this model, further flow cytometric analyses demonstrated high expression of IL-1β and CCL3 in this CD11b^+^CD44^hi^ MNP subset and showed that expression of these proinflammatory mediators in CD44^hi^ MNPs was increased in the absence of MAIT cells (Fig. 5k). A similar trend of MAIT cell-dependent regulation of these myeloid cell-expressed proinflammatory cytokines identified in Fig. 5g was observed in total mRNA expression in the kidney cortex (Supplementary Fig. 9). Finally, direct interaction of CD11b^+^ cells with PLZF^+^RORc-GFP^+^ MAIT cells in the periglomerular infiltrates was confirmed by immunofluorescence analyses (Fig. 5l). In summary, these findings supported the interactome-based working model in which MAIT cells may mediate suppression of proinflammatory MNP functions in cGN.

### Attenuation of cGN by MAIT cells depends on the CXCL16–CXCR6 axis

**Fig. 6:**
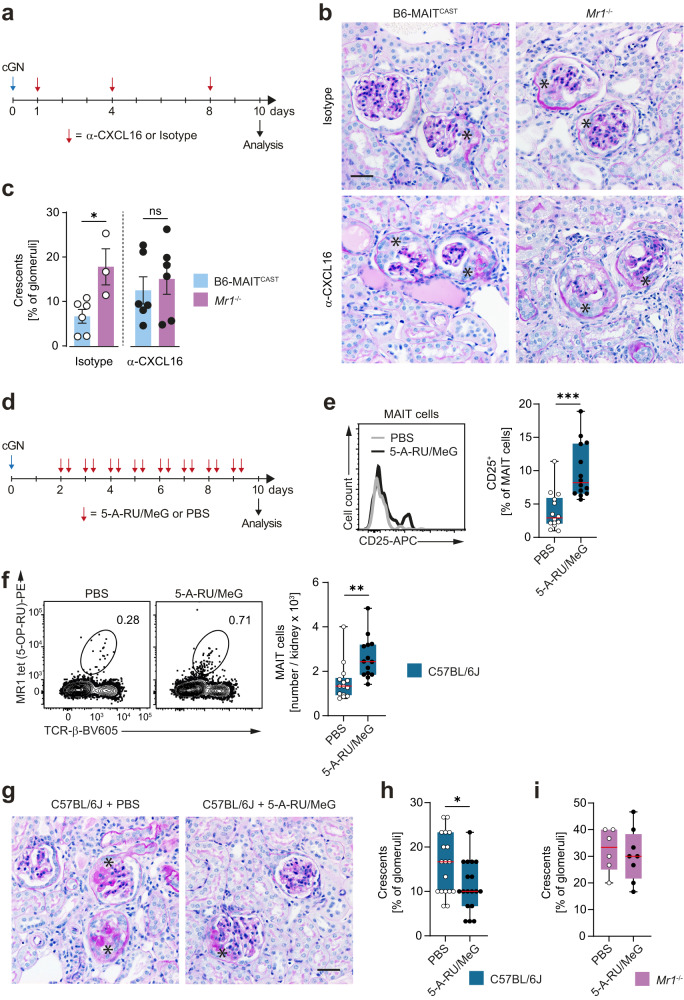
CXCL16 blockade abrogates protective effect of mucosal-associated invariant T (MAIT) cells and pharmacological MAIT cell activation ameliorates experimental crescentic glomerulonephritis (cGN). **a** Schematic representation of experimental setup for α-CXCL16 treatment in cGN. **b** Representative photomicrographs of periodic acid-Schiff-stained kidney sections from isotype- and α-CXCL16-treated B6-MAIT^CAST^ and *Mr1*^−/−^ mice on day 10 of cGN. **c** Quantification of crescent formation in the respective groups of mice (B6-MAIT^CAST^ + Isotype *n* = 6, *Mr1*^*−/−*^ + Isotype *n* = 3, B6-MAIT^CAST^ + α-CXCL16 *n* = 6, *Mr1*^*−/−*^ + α-CXCL16 *n* = 6, *n* represents biologically independent animals for each group). Data are pooled from two independent experiments. **d** Schematic representation of experimental setup for 5-A-RU/MeG treatment in cGN. **e** Representative histogram and quantification of CD25 expression in PBS- and 5-A-RU/MeG-treated C57BL/6J wild type mice with cGN. (day 10, PBS *n* = 14, 5-A-RU/MeG *n* = 14, *n* represents biologically independent animals for each group). **f** Representative flow cytometry plots and quantification of MAIT cells in kidneys of PBS- and 5-A-RU/MeG-treated C57BL/6J wild type mice with cGN (day 10, PBS *n* = 14, 5-A-RU/MeG *n* = 14, *n* represents biologically independent animals for each group). Data in (**e** and **f**) are pooled from three independent experiments with similar results. **g** Representative photomicrographs of periodic acid-Schiff-stained kidney sections from PBS- and 5-A-RU/MeG -treated C57BL/6J wild type on day 10 of cGN. Quantification of crescent formation in PBS- and 5-A-RU/MeG-treated C57BL/6J wild type mice (**h**) and *Mr1*^−/−^ mice (**i**) with cGN (wild type day 10, PBS *n* = 18, 5-A-RU/MeG *n* = 18, *Mr1*^−/−^ day 10, PBS *n* = 6, 5-A-RU/MeG *n* = 8; *n* represents biologically independent animals for each group). Data in (**f**) are pooled from four independent experiments and data in (**i**) are pooled from two independent experiments. Asterisks mark glomerular crescents. Scale bar represents 20 µm. Circles represent individual animals and bars represent mean ± SEM (*p < 0.05, **p < 0.01, ***p < 0.001 in two-sided Student’s *t* test). Source data are provided as a Source Data file.

To further validate this working model, we decided to interrupt the CXCR6–CXCL16 axis in mice with cGN by injection of a CXCL16-blocking antibody^41^ (experimental scheme in Fig. 6a). As described above (see Fig. 4), isotype-treated B6-MAIT^CAST^ mice were protected from cGN and developed only mild disease, while isotype-treated *Mr1*^−/−^ mice showed aggravated disease. CXCL16 blockade increased the percentage of crescents in B6-MAIT^CAST^ mice to a level similar to that observed in *Mr1*^−/−^ mice (Fig. 6b, c), confirming that the CXCL16-CXCR6 interaction is necessary for MAIT cell-mediated protection against cGN. We previously reported that cGN is aggravated in CXCR6^−/−^ mice, which we had attributed to a protective effect of CXCR6^+^ NKT cells^40^. To clarify to which extent these cells were involved, we antagonized CXCL16 also in MAIT cell-deficient *Mr1*^−/−^ mice with cGN. In these experiments, tissue damage was not significantly higher in α-CXCL16-treated *Mr1*^−/−^ mice compared to α-CXCL16-treated B6-MAIT^CAST^ mice and comparable to the level of *Mr1*^−/−^ mice without CXCL16 blockade (Fig. 6b, c), which might indicate that MAIT cells mediated the major part of the protective effect of the CXCR6–CXCL16 axis.

### Pharmacological MAIT cell activation mitigates crescentic glomerulonephritis

Finally, we asked whether our findings allow for therapeutic translation. To this end, we decided to activate and expand MAIT cells in vivo by treating mice with the synthetic MAIT TCR ligand, 5-amino-6-D-ribitylaminouraci (5-A-RU)/methylglyoxal^42,43^. We modified and optimized existing protocols for chemical synthesis of this ligand by devising new purification protocols that showed a yield of 5-A-RU in 72% purity over six steps (see Supplementary Methods) and allowed the use of commercially available chemicals. This protocol proved robust and reliable and the obtained yield compared favorably to previous approaches^44–46^. We injected our 5-A-RU/MeG compound at a previously titrated concentration of 100 nmol per day into C57BL/6 mice (Supplementary Fig 10), starting from day 2 after induction of cGN, to mimic the clinically more relevant situation of treating ongoing disease rather than treating disease initiation (experimental scheme in Fig. 6d). This treatment activated intrarenal MAIT cells, as indicated by their increased expression of the activation marker CD25, and expanded their numbers by 2–3-fold (Fig. 6e, f). We noted a moderate, but significant improvement of histopathological tissue injury in C57BL/6 wild type mice with cGN (Fig. 6g, h), supporting the principal idea that MAIT cell expansion might be protective in experimental cGN and might therefore represent a promising therapeutic strategy in cGN. Of note, the protective effect of 5-A-RU/MeG application (same experimental setup as in Fig. 6d) was not observed in *Mr1*^−/−^ mice, arguing against potential off-target effects of 5-A-RU/MeG or its metabolic products (Fig. 6i). However, the protection mediated by application of 5-A-RU/MeG to B6 wild type mice was not as robust as that observed in MAIT cell-enriched B6-MAIT^CAST^ mice when compared to B6 mice (see Fig. 4) which might be explained by a potential 5-A-RU-induced activation of proinflammatory MAIT17 activity^47^ that might partially counteract their protective effect in cGN.

## Discussion

MAIT cells have recently gained much attention in inflammatory and infectious diseases of many organs, but their role in kidney disease has not been addressed yet. Here we report the first detailed single cell transcriptomic profiling of MAIT cells in a peripheral organ, the kidney, under noninflamed conditions and during a non-infectious IMID. Previous studies had used flow-cytometry to describe MAIT cells with a tissue-resident phenotype in human kidney tissue^27–29^. We identified intrarenal MAIT cells bearing an activated MAIT17 phenotype in human kidney tissue from healthy individuals and from patients with ANCA-GN^31^. This contrasts the situation in the lung, where both MAIT17 and MAIT1 subsets were detected in the bronchoalveolar lavage fluid of lungs from patients with bacterial and COVID19 pneumonia^32,48^. In most mouse tissues, including secondary lymphoid organs, liver, lung, colon, and skin, MAIT17 cells are abundant, but MAIT1 cells are also present, and both subsets share a tissue-resident transcriptomic fingerprint with other innate-like T cells, such as NKT1 and NKT17 cells^49^. Also in kidneys of MAIT cell-enriched B6-MAIT^CAST^ mice, we found a population of MAIT cells very similar in size and composition to that of the human kidney, again with a large majority being RORγt^+^ IL-17A-producing MAIT17 cells bearing a tissue-resident phenotype.

Single cell transcriptomic profiling of intrarenal MAIT cells also provided insights into potential effector functions of these intrarenal MAIT17 cells in experimental murine cGN. We noted that the MAIT17 cell subset was expanded and activated during kidney inflammation. Interestingly, in addition to producing proinflammatory IL-17A, this subset was characterized by high expression of potential anti-inflammatory mediators, such as CTLA4, PD-1, and TGF-β. Indeed, several gain- and loss-of-function experiments, indicated that MAIT cells might attenuate tubulointerstitial inflammation and crescent formation in experimental cGN and improve the clinical outcome. Previous studies reported that MAIT cells attenuated tissue damage in some IMID models, e.g., experimental autoimmune encephalomyelitis^24^ and type 1 diabetes in non-obese diabetic mice^25^. By contrast, MAIT cells aggravated disease in other IMID models, such as collagen-induced arthritis^50^ and the *FcgR2b*^−/−^*Yaa* lupus model^30^. These variable outcomes highlight the context- and tissue-dependent role of MAIT cells in experimental models and the functional role of MAIT cells in kidney inflammation had not been studied so far.

The mechanisms proposed for these divergent roles included for example regulation of systemic B cell^24,30^ or T cell responses^30,50^, or altered gut barrier function^25^. In our study, MAIT cells did not act systemically as in these reports, but locally within the kidney. To identify these local mechanisms, we performed an unsupervised immune cell interactome analysis on our single-cell transcriptome dataset of intrarenal immune cells as recently described^32^. This revealed potential interactions of activated MAIT17 cells with a proinflammatory MNP subset. Three of the top 20 interaction partners were immunosuppressive and have previously been described to ameliorate cGN: CTLA4–CD86, TGF-β1–TGF-βR1, and CXCR6–CXCL16^35,39,40^. We hypothesized that CXCR6^+^ MAIT cells were attracted to CXCL16-producing inflammatory MNPs and employ CTLA4 and/or TGF-β1, and potentially other mediators, to dampen their activation, a step previously shown to be essential in the progression of experimental cGN^34,51^. This model is consistent with studies in other organs, reporting interaction of MAIT cells with proinflammatory MNPs^26,52^, with a study reporting that the CXCR6–CXCL16 axis affects MAIT cell migration^53^, and with the expression of CTLA4^54^ and TGF-β^55^ by MAIT cell subsets. We provided further support for this model by demonstrating that intrarenal MAIT cells indeed colocalize with CD11b^+^ MNP in periglomerular infiltrates and by showing that MNP displayed an activated and proinflammatory phenotype when MAIT cells were absent, as predicted by our interactome analysis.

We next aimed to provide functional evidence for a protective role of the MAIT–MNP interaction. As described above, the interactome predicted multiple candidate suppressive pathways, suggesting that blocking just one of these interaction pairs might not be effective enough for significant changes. Instead, we reasoned that interrupting MAIT–MNP colocalization by blocking the CXCR6–CXCL16 axis might be able to target all downstream candidate suppressive pathways. Indeed, blocking this axis was sufficient to impair renal tissue protection in the presence of increased MAIT cell numbers (B6-MAIT^CAST^ mice), supporting our hypothesis. Other protective cells in cGN, especially NKT cells, might contribute to the protective effect of the CXCR6–CXCL16 pathway^40^, but these were not the major players, as indicated by the lack of an effect of the CXCL16 blockade in MAIT-cell-deficient mice.

In summary, we show in two ways that increasing MAIT cell numbers attenuates disease and in two other ways that decreasing their numbers or function aggravates disease and support it with single cell transcriptomics. A limitation of our study is the infeasibility to support the protective effect of MAIT cells by adoptive transfer experiments. Adoptive transfers of MAIT cells have been successfully performed in immunocompromised hosts, e.g., *Cd3*^−/−^^56^ or *Rag*^−/−^*Il2rg*^−/−^^57^, in which our cGN model does not develop. In addition, strategies for transfer of MAIT cells into MAIT cell-deficient *Mr1*^−/−^ mice have recently been reported for investigating brain homeostasis^58^, cancer^59^, and bacterial infection^60^. However, despite extensive experimental attempts, similar transfer strategies were insufficient to reconstitute kidney MAIT cell populations in *Mr1*^−/−^ mice in our hands. Therefore, identifying the mechanism for MAIT cell-mediated renal tissue protection would require developing genetic tools to selectively delete potential suppressive mediators (e.g., CTLA4, TGF-β) from MAIT cells in future studies.

Based on these mechanistic findings, we speculated that pharmacological MAIT cell activation might be a therapeutic approach in cGN. Several synthetic MAIT cell ligands have been described, and 5-A-RU/MeG is the most widely studied and potent of them^43^. To obtain the considerable doses of that ligand for treating mice twice daily over extended periods, we optimized existing methods to improve yield and purity. Treatment with our ligand indeed ameliorated the hallmark histological features of cGN in wild type mice, but not in *Mr1*^−/−^ mice, ruling out potential off-target effects of 5-A-RU/MeG or its metabolic products. Despite this proof of concept, clinical parameters for kidney function were less improved than in B6-MAIT^CAST^ mice, which may be explained by the very short half-life of the ligand, limiting its protective activity in vivo^61^. Another explanation for the limited protective activity of the ligand in vivo, might be the activation of proinflammatory properties of MAIT17 cells by TCR stimulation, which would be consistent with previous studies showing increased IL-17A production in other organs and in vitro after 5-A-RU/MeG treatment^47,53^. In cGN, IL-17 can aggravate the early phases of disease by recruiting neutrophils to the inflamed kidney^62,63^. Several approaches to enhance TCR ligand-mediated MAIT cell activation in vivo by adding proinflammatory cytokines, e.g., IL-23, or TLR agonists have been reported^64,65^. However, these inflammatory mediators are potent aggravating factors in cGN and other autoimmune models^66,67^, precluding their use to enhance protective MAIT cells functions in this context.

These considerations argue against 5-A-RU/MeG alone as a therapeutic option, but it might be effective in a combination therapy with established immunosuppressive medications. Moreover, distinct TCR ligands for NKT cells have been produced that selectively induce a Th1- or Th2-polarization bias^68^. The hypothetical development of such functionally biased MAIT cell TCR ligands that do not induce proinflammatory MAIT17 activity, but instead promote their anti-inflammatory properties, warrants further study.

In summary, we demonstrate here that a MAIT17 population with anti-inflammatory properties resides in the murine and human kidney and gets activated during cGN. Transcriptomic profiling, interactome analyses and in vivo functional validation of the predicted pathways suggest interaction of MAIT17 cells with proinflammatory myeloid cell populations in the kidney via the CXCR6–CXCL16 axis, resulting in suppression of myeloid cell tissue destructive capacity. The pharmacological targeting of MAIT cells, described here, might help to develop MAIT-cell-based interventions for the treatment of cGN and perhaps of further IMIDs. Such therapies based on vitamin derivatives might synergize with established immunosuppressive and anti-inflammatory therapies.

## Methods

### Human material

Human studies were approved by the local ethics committee of the chamber of physicians in Hamburg (Ethik-Kommission der Ärztekammer Hamburg, approval number PV 5822), and were conducted in accordance with the ethical principles stated by the Declaration of Helsinki. Human kidney cortex specimen and blood samples were obtained from patients that underwent partial nephrectomy because of suspected renal carcinoma after written informed consent. Human samples were provided in an anonymized fashion, precluding the analysis of age- and gender-related effects. Samples of macroscopic healthy, tumor-free kidney cortex were excised from the residual tissue that was not needed for diagnostic purposes. Further blood samples were drawn from healthy volunteers after written informed consent.

### Animals

B6.129P2-Mr1^tm1Gfn^ (*Mr1*^*−/−*^*)*^6^ and B6(Cg)-Mait^hi^Rorc^tm2Litt^ (B6-MAIT^CAST^)^33^ mice were obtained from Olivier Lantz (Paris, France) and were transferred to and bred in the animal facility of the University Medical Centre Hamburg-Eppendorf, or in the central animal facility of the University Clinic of Bonn. In all experiments, adult (>8 week-old), sex- and age-matched mice were used. A total of 273 mice (27% female) were used in the experiments presented in Figs. 2–6 and in the Supplementary Information. We preferentially used male mice, as we observe a higher variability with female mice in the cGN model. Although gender-related effects were not formally addressed in this study, the main results were comparable in all experiments, regardless of which sex was used. For detailed information regarding the specific strain, sex, age, and number of all animals in every experiment please refer to the Figure Legends, the Supplementary Information, and the Source Data file provided with this study. All animals were raised under specific pathogen-free conditions in a facility at temperatures of 21–24 °C with 40–70% humidity on a 12 h light/12 h dark cycle. Standard chow and water were provided ad libitum. Experimental mice and controls were bred separately in the same animal facility. Euthanasia was performed by cardiac puncture under deep isoflurane anesthesia. All animal experiments were performed according to national and institutional animal care and ethical guidelines and were approved by the local committees (approval number N020/2022, Behörde für Gesundheit und Verbraucherschutz, Freie und Hansestadt Hamburg).

### Induction of experimental crescentic glomerulonephritis and functional analyses

Crescentic glomerulonephritis (cGN) was induced by intraperitoneal injection of nephrotoxic sheep serum (100–200 µl, depending on batch) in adult mice^69^. Of note, severity of the cGN model varies with different batches of nephrotoxic sheep serum. For urine sample collection, mice were housed in metabolic cages for 5 h. Urinary albumin excretion was determined by standard ELISA (Mice-Albumin Kit; Bethyl Laboratories, Montgomery, TX). Urinary creatinine levels were measured with the Creatinine Jaffé Fluid (Hengler Analytik, Steinbach, Germany). Blood urea nitrogen (BUN) was analyzed by standard laboratory procedures.

### Synthesis of 5-A-RU

Benzyl amine was added to a suspension of d-ribose in methanol. After hydrogenation with H_2_ over PtO_2_, filtration and concentrating, ethyl acetate was added to crystalize *N*-benzyl-d-ribityl amine. After separation of the crystals, they were again recrystallized using a solution of ethyl acetate/ethanol (3:1) to receive *N*-benzyl-d-ribityl amine. The solution of *N*-benzyl-d-ribityl amine in methanol was hydrogenated with H_2_ over 10% Pd/C and dried after filtration to obtain an oil of d-ribityl amine. This was boiled together with 6-chlorouracil in H_2_O under reflux. Ethanol was added to the concentrated solution and the obtained gummy mass was washed and dried. This product was then dissolved in hot water and cooled to 5 °C. NaNO_2_ as well as a solution of acetic acid was added. After evaporating the product to dryness, it was dissolved in 0.15 m NH_3_ and placed on a Dowex 1-X8 200-400 (Cl-form) column. The column was washed with H_2_O and 0.01 m formic acid. 0.1 m formic acid was placed on the anion exchange resin and the red percolate was collected and concentrated. The residue was crystallized and nitroso-6-d-ribitylaminouracil was obtained. After dissolving nitroso-6-d-ribitylaminouracil in degassed, pure water it was hydrogenated with H_2_ over 10%-Pd/C, filtered over a flame-dried plug of Celite^©^ placed on a flame-dried filter paper and washed with degassed, pure water. The yellow filtrate was lyophilized under reduced pressure obtaining pure yellow solid 5-A-RU. 5-A-RU was then dissolved in DMSO and diluted in PBS for in vivo studies.

### Treatment with 5-A-RU/MeG

For titration of optimal dosing of the MAIT cell-stimulating ligand, increasing concentrations of 5-A-RU/MeG were applied i.p. twice daily from day 2 to day 9 after induction of cGN (total dose per day: 25, 50, 100, 200 nmol). For therapeutic administration, C57BL/6 wild type and *Mr1*^−/−^ mice were injected with 50 nmol 5-A-RU/MeG intraperitoneally twice daily (total dose per day: 100 nmol) from day 2 to day 9 after induction of cGN. Prior to injection, 5-A-RU was mixed with methylglyoxal (Sigma-Aldrich) in a 1:1 ratio. Controls were injected with the same volume of PBS.

### Anti-CXCL16 and anti-MR1 treatment

For anti-CXCL16 treatment, mice were injected intraperitoneally (i.p.) with 300 µg anti-CXCL16 (R&D Systems, clone 142417) or Rat IgG2A as isotype control (R&D Systems, clone 20102) on day 1. On day 4 and 8 100 µg of anti-CXCL16 or isotype control was injected intraperitoneally. For anti-MR1 treatment, α-MR1 antibody (Biolegend, clone 8F2.F9) was administered i.p. 1 day before and on days 3 and 7 after induction of the cGN model at a concentration of 200 µg per injection. IgG1k isotype (200 µg per injection, Biolegend, clone MOPC-21) was used as control and injected in the same manner.

### Cell isolation

For isolation of renal leukocytes, mouse kidneys were cut into small pieces and were enzymatically digested in complete medium (RPMI 1640, 10% Fetal Bovine Serum, 1% HEPES, 1% Penicillin/Streptomycin; all Gibco, Thermo Fisher Scientific, USA) supplemented with Collagenase D (0.4 mg/ml, Roche, Basel, Switzerland) and DNase I (100 µg/ml, Roche) for 45 min at 37 °C while rotating on a MACSmix® tube rotator (Miltenyi, Bergisch Gladbach, Germany). After further dispersion with the gentleMACS® dissociator (Miltenyi), leukocytes were purified by Percoll gradient centrifugation (37.5%) (GE Healthcare, Chicago, USA) and further enriched by subsequent erythrocyte lysis with ammonium chloride. After filtration through a 50-µm strainer, cell suspension was ready for further analyses. For leukocyte isolation from the lung, tissue was cut into small pieces and digested in complete medium supplemented with Liberase (0.42 mg/ml, Roche) and DNase I (100 µg/ml, Roche) for 45 min at 37 °C on the MACSmix®tube rotator. The cell suspension was further purified by mashing through a 70-µm strainer, followed by a Percoll gradient centrifugation (37.5%) and subsequent erythrocyte lysis as described above. Leukocytes from the liver were isolated by mashing the tissue through a 100-µm strainer and further purified by Percoll gradient centrifugation (37.5%) and erythrocyte lysis. For the isolation of splenocytes, the spleen was mashed through a 70-µm strainer and a subsequent erythrocyte lysis further enriched the splenocyte population. For the isolation of PBMCs from human patients, blood was drawn into BD Vacutainer® CPT tubes and centrifuged according to the manufacturer’s instructions. Tissue processing and leukocyte isolation of human kidney samples were performed similar to the mouse protocol. 0.5–1 g of kidney tissue was used for enzymatic digestion with Collagenase D (0.4 mg/ml, Roche) and DNase I (100 µg/ml, Roche) as described above.

### Flow cytometry

To characterize leukocyte subsets, cell suspensions were stained with fluorochrome-coupled antibodies against CD45 (1:200, Biolegend, clone 30-F11), CD3 (1:50, BD Bioscience, clone 145-2C11), CD4 (1:200, Biolegend, clone RM4-5), CD8 (1:400, Biolegend, clone 53-6.7), TCR-β (1:200, Biolegend, clone H57-597), TCR-γδ (1:100, Biolegend or BD Bioscience, clone GL3), CD11b (1:1000, Biolegend, clone M1/70), Ly6G (1:200, Biolegend, clone 1A8), B220 (1:400, Biolegend, clone RA3-6B2), CD69 (1:100, Biolegend, clone H1.2F3), CD127 (1:100, Biolegend, clone A7R34), CD103 (1:50, Biolegend, clone 2E7) CD44 (1:100, Biolegend, clone IM7), CXCR6 (1:100, Biolegend, clone SA051D1), CD25 (1:100, Biolegend, clone QA19A49), and IL18R (1:25, Miltenyi or Biolegend, clone REA947). Human cell suspensions were stained with fluorochrome-coupled antibodies against CD45 (1:200, Biolegend, clone HI30), CD3e (1:200, Biolegend, clone OKT3), γδTCR (1:25, Biolegend, clone B1), CD161 (1:50, Biolegend, clone HP-3G10), CD127 (1:200, Biolegend, clone A019D5), CD69 (1:100, Biolegend, clone FN50), IL-18R (1:200, Biolegend, clone H44), RORγt (1:40, BD Biosciences, clone Q21-559). Nonspecific staining was prevented by incubation with 10% normal mouse serum (Jackson ImmunoResearch Laboratories, West Grove, USA). Dead cells were stained using LIVE/DEAD Fixable Read Dead Stain Kit (Invitrogen) or Zombie Dye (Biolegend, San Diego, USA). For staining of circulating cells, fluorochrome-coupled CD45.2 antibody (2 µg per animal) were intravenously injected 5 min prior to euthanasia. For intracellular staining of cytokines, isolated leukocytes were stimulated with phorbol 12-myristate 13-acetate (50 ng/ml, Sigma-Aldrich) and ionomycin (1 µg/ml, Calbiochem) in the presence of brefeldin A (10 µg/ml, Sigma) for 2.5 h at 37 °C. After subsequent surface staining, cells were fixed with formalin (3.7%, Sigma-Aldrich), permeabilized with IGEPAL® CA-630 (0.1%, Sigma-Aldrich), and stained with combinations of antibodies against IL-1β (1:100, Invitrogen, clone NJTEN3), CCL3 (1:100, Invitrogen, clone DNT3CC), CD152 (1:100, Biolegend, clone UC10-4B9), CXCL16 (1:200, BD Bioscience, clone 12-18), IL-17A (1:200, BioLegend, clone TC11-18H10.1), IFN-γ (1:200, BioLegend, clone XMG1.2), and IL-4 (1:200, BioLegend, 11B11). For staining of MAIT cell cytokine production, the above mentioned protocol was optimized. In brief, stimulation was performed with a lower phorbol 12-myristate 13-acetate concentration (10 ng/ml, Sigma) and unchanged concentrations of ionomycin (1 µg/ml, Calbiochem) and brefeldin A (10 µg/ml, Sigma) for 4 h at 37 °C. After 2 h, the fluorochrome-coupled 5-OP-RU MR1-tetramer was added to the medium. Next, the surface staining was performed as described above. To maintain GFP reporter signal after permeabilization for intracellular and intranuclear staining, cells were fixed with formalin (3.7%, Sigma-Aldrich) for 15 min at room temperature and subsequently washed with Perm/Wash buffer (Transcription Factor Staining Buffer Set, eBiosciences). Intracellular antibodies against IL-17A (1:200, Biolegend, clone TC11-18H10.1), IFN-γ (1:200, Biolegend, clone XMG1.2), and IL-4 (1:200, Biolegend, clone 11B11) were diluted in Perm/Wash buffer and cells were incubated over night at 4 °C in the dark. Intranuclear transcription factor staining of MAIT cells was performed according to the same protocol without prior restimulation by using antibodies against GATA-3 (1:100, BD Bioscience, clone L50-823), T-bet (1:100, Biolegend, clone 4B10), and RORγt (1:200, BD Bioscience, clone Q31-378). MAIT cell-specific human 5-OP-RU-loaded (1:800, lot 39237, 2018), 6-FP-loaded (1:800, lot 39238, 2018), mouse 5-OP-RU-loaded (1:200, lot 50922, 2020), and 6-FP-loaded (1:200, lot 50921, 2020) MR1-tetramers were provided by the NIH Tetramer Core Facility. Absolute CD45^+^ cell numbers in cell suspension were determined by staining with fluorochrome-coupled anti-CD45 combined with cell count beads (Countbright®, Invitrogen). All samples were acquired on a LSRII or FACSymphony flow cytometry (both BD Biosciences) and analyzed with the FlowJo Software (Treestar Inc.).

### Flow cytometry-based cell sorting

MAIT cells from naïve murine kidneys, as well as on days 1 and 9 after induction of cGN were defined as CD11b^−^CD19^−^B220^−^TCR-γδ^−^CD3^+^TCR-β^+^Mr1 tet (5-OP-RU)^+^ and sorted with a FACSAria™ Fusion Cell Sorter (BD Biosciences). On day 9 of cGN, renal CD45^+^ non-MAIT cells were sorted in parallel. The gating strategy for cell sorting is depicted in Supplementary Fig. 3B.

### scRNA sequencing of murine leukocytes

Single-cell libraries of sorted cells were generated with the 10x Genomics Chromium Single Cell 5’v1.1 reagents kit according to the manufacturer’s protocol. The libraries were sequenced on Illumina NovaSeq 6000 with 150 base pairs and paired-end configurations. Transcriptomic data from Mr1 tet(5-OP-RU)^+^ MAIT cells (day 1 and day 9 after induction of cGN) and CD45^+^ non-MAIT cells (day 9 after induction of cGN) were clustered separately using the FindIntegrationAnchors function and IntegrateData function of Seurat. After exclusion of low quality cells (<200 genes expressed), low expressed genes (expressed in less than 3 cells) and cells with upregulated mitochondrial genes (more than 5% of all reads), unsupervised clustering was performed by standard Seurat workflow. Uniform manifold approximation and projection for dimension reduction (UMAP) calculated for the first 30 principal components was used for dimensionality reduction and plotting. Clusters were assigned using Seurats FindClusters function with a resolution of 0.2 for MAIT cells and 0.5 for non-MAIT cells. Genes for the murine MAIT cell activation score were obtained from the dataset of Hinks et al.^22^ by extracting significantly upregulated genes from the differential expression analysis between murine MAIT cells in acute infection and uninfected CD8^+^CD44^lo^CD62^hi^ cells and generated by using the AddModuleScore function with default parameters. Differential expression analyses between cell clusters was performed with the FindAllMarkers function of Seurat. Interactome analysis was performed using CellPhoneDB (https://www.cellphonedb.org/). The normalized expression matrices of murine MAIT cells and other CD45^+^ were merged and analyzed. Since the database works with human genes, mouse genes were translated into their human homologs. Mouse genes without a human homolog were omitted. For analysis, protein-protein interactions with more than two proteins were excluded. We used the layout.fruchterman.reingold function of the R package iGraph in combination with the plot function to plot the number of significant interactions between the clusters.

### Analysis of published scRNAseq dataset

Human MAIT cell-defining genes were determined in a single-cell sequencing dataset from blood of COVID-19 and bacterial pneumonia patients^32^ using the FindMarkers function of the R package Seurat (v.4.0.0). The 20 differentially expressed genes with the lowest adjusted *p* value were then used as MAIT cell score in a dataset of sorted kidney CD3^+^ T cells of patients with ANCA-GN and healthy controls^31^. For Fig. 1c, cells with a MAIT cell score above 0.45 were highlighted. For the human MAIT cell activation score, significantly upregulated genes of in vitro 5-OP-RU-activated human MAIT cells compared to naïve MAIT cells were used from a dataset of Hinks et al.^22^. The scores were generated using AddModuleScore function with default parameters of the R package Seurat.

### Real-time RT-PCR

Total RNA of the renal cortex was prepared according to standard laboratory methods. After reverse transcription to cDNA, real-time PCR was performed for 45 cycles (initial denaturation at 95 °C for 10 min, followed by cycles of denaturation at 95 °C for 15 s, and primer annealing and elongation at 60 °C for 1 min) with 1 µl of cDNA samples in the presence of 0.5 µl of specific murine primers (CXCL2 Mm00436450_m1; CCL3 Mm00441259_g1; Il1b Mm00434228_m1; all Thermo Fisher Scientific). TaqMan® Gene Expression Assays and a StepOnePlus Real-Time PCR system (both Thermo Fisher Scientific) were used for quantification of the housekeeping gene (*Hprt1*) and the genes of interest. All samples were run in duplicates.

### Histopathology and immunohistochemistry

Formalin-fixed, paraffin-embedded kidney sections were stained with Periodic acid-Schiff’s (PAS) reagent according to standard laboratory procedures. Crescent formation in the cGN model was assessed in 30 glomeruli per mouse. For quantification of interstitial neutrophil cell numbers, paraffin-embedded sections were stained with an antibody directed against the neutrophil marker GR-1 (Ly6 G/C) (1:10,000, NIMP-R14; Hycult Biotech, The Netherlands) and developed with a polymer-based secondary antibody alkaline phosphatase kit (POLAP; Zytomed, Berlin, Germany). GR-1^+^ cells were counted in ten low-power fields (original magnification ×200) per section. All histological quantifications were performed in a blinded fashion.

### Immunofluorescence

For immunofluorescence staining, 2.5-μm thick paraffin sections were heated at 98 °C for 20 min in 0.05% citraconic acid anhydride antigen retrieval buffer (pH 7.4). Unspecific binding was blocked with 5% horse serum (Vector Laboratories, USA) and 0.05% triton-X100 PBS. The sections were then incubated with rabbit-anti-GFP (1:1000, Abcam, United Kingdom) and goat-anti-PLZF (1:500, R&D Systems, USA) in 5% horse serum overnight at 4 °C. The staining was visualized using AF488- (anti-goat) and Cy3- (anti-rabbit) conjugated secondary antibodies (1:100; Jackson ImmunoResearch Laboratories, USA) incubated for 30 min at room temperature. For co-localization studies, rat-anti-CD11b (Clone M1/70, Biolegend) with visualization by Cy5 (1:50) was included. DNA was counterstained with Hoechst (Thermo Fisher Scientific, USA). Images of the stained slides were acquired with an LSM 510 Meta confocal microscope using the LSM software (Zeiss, Jena, Germany).

### Statistics and reproducibility

Selection of sample size for animal experiments in the cGN model was based on long-standing experience with the variability of the model. No statistical method was used to predetermine sample size. Animals were randomly assigned to different treatment groups. All animal experiments were performed at least twice with similar results. No data were excluded from the analyses of animal outcome experiments. The investigators were not blinded to allocation during experiments, but all histology samples for assessment of cGN outcome were analyzed in a blinded fashion. The Student’s *t* test was used for comparison between two groups. In case of three or more groups, one-way ANOVA was used followed by a post hoc analysis with Newman-Keuls test for multiple comparisons. A *p* value of <0.05 was considered to be statistically significant.

### Reporting summary

Further information on research design is available in the Nature Portfolio Reporting Summary linked to this article.

### Supplementary information

Supplementary Information

Reporting Summary

### Source data

Source Data

## Data Availability

The sequencing data generated in this study have been deposited to the National Center for Biotechnology Information Gene Expression Omnibus (GEO) and are accessible through the GEO Series accession number GSE245302. The sequencing data used in Fig. 1 have been published before^31^. All other data generated in this study are provided in the Supplementary Information and/or Source Data file. All other relevant data are available from the corresponding author on request. Source data are provided with this paper.
