# Supplementary material for: Mucosal-associated invariant T cells contribute to suppression of inflammatory myeloid cells in immune-mediated kidney disease
Source: Nat Commun. 2023 Nov 15;14:7372. doi: 10.1038/s41467-023-43269-0 (PMC10651937; doi:10.1038/s41467-023-43269-0)
Supplement: Supplementary file 1 — Supplementary Information [file 41467_2023_43269_MOESM1_ESM.pdf]

Supplementary Figure 1

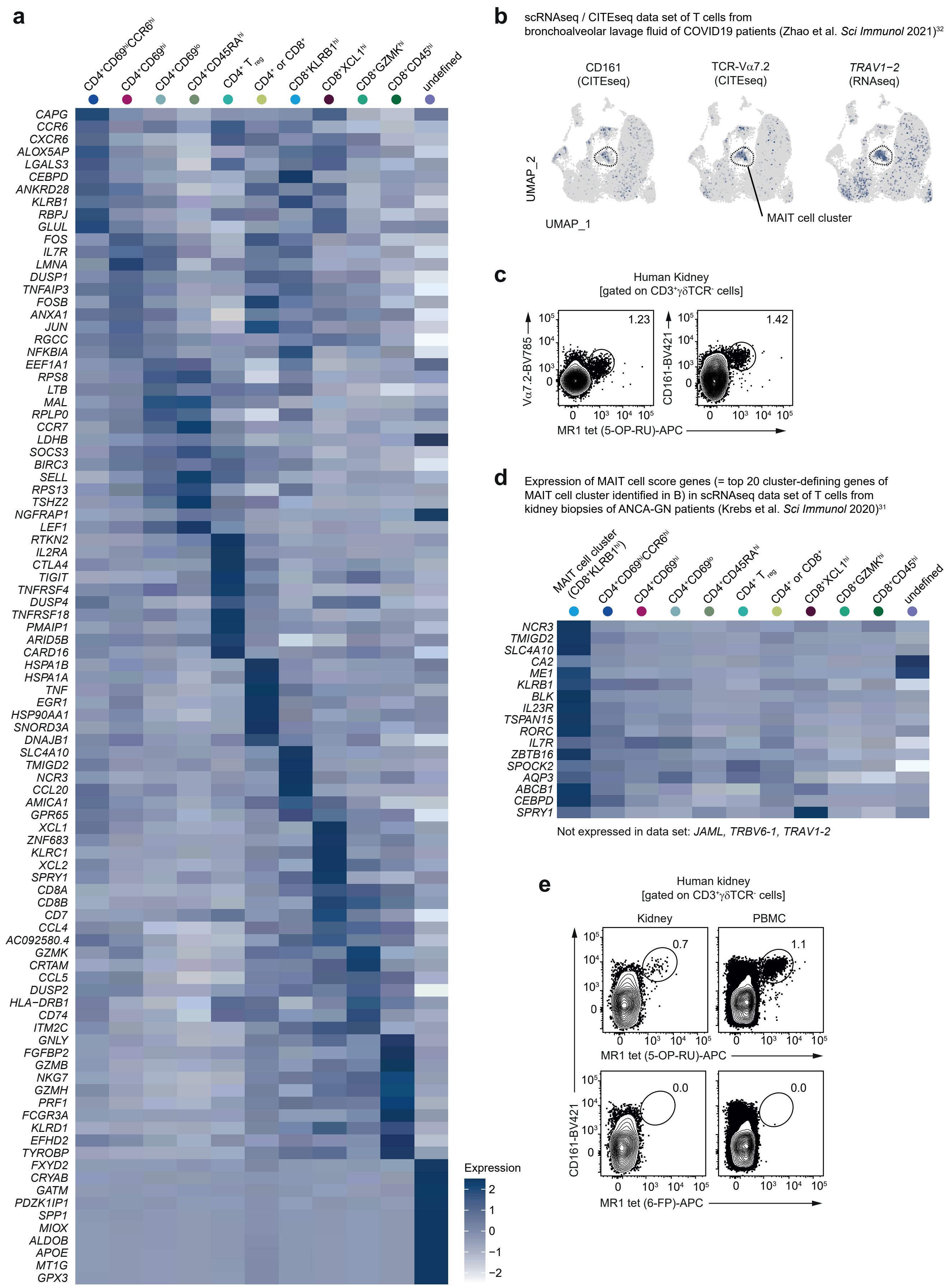

**Figure S1: Expression of cluster-defining genes and identification of Mucosal-associated invariant T (MAIT) cell-specific gene expression module (A)** Normalized expression heatmap of the top ten cluster-defining genes in the eleven cell clusters identified by single-cell RNA sequencing (scRNAseq) of T cells from kidneys of anti-neutrophil cytoplasmatic antibody-associated glomerulonephritis (ANCA-GN) patients and controls. **(B)** Uniform Manifold Approximation and Projection representation of a published data set of T cells isolated from the bronchoalveolar lavage fluid of Coronavirus Disease 2019 (COVID19) patients in which $CD161^{+}$TCR-$V\alpha7.2^{+}$ *TRAV1-2*-expressing MAIT cells were identified by a combined scRNAseq / CITE-seq (CITE = cellular indexing of transcriptomes and epitopes by sequencing) approach[32]. **(C)** Representative flow cytometry plots of CD161 and TCR-Vα7.2 surface expression on MR1 5-OP-RU-loaded tetramer-positive MAIT cells in unaffected kidney tissue of a tumor nephrectomy patient **(D)** Normalized expression heatmap of the uncurated list of MAIT cell score genes (= top 20 cluster-defining genes of the MAIT cell cluster identified in the COVID19 data set in B) in the eleven clusters of the scRNAseq data set of T cells from kidney biopsies of ANCA-GN patients[31] (Figure 1). **(E)** Representative flow cytometry plots of paired kidney and blood samples from a tumor nephrectomy patient stained with MR1 tetramers loaded with either the specific MAIT-T cell receptor (TCR) ligand 5-OP-RU or the non-TCR-binding control molecule 6-FP. Gating strategy is specified in brackets and numbers indicate the percentage of cells in the gate.

# Supplementary Figure 2

A-C: scRNAseq / CITEseq data set of T cells from
bronchoalveolar lavage fluid of COVID19 patients (Zhao et al. *Sci Immunol* 2021)[32]

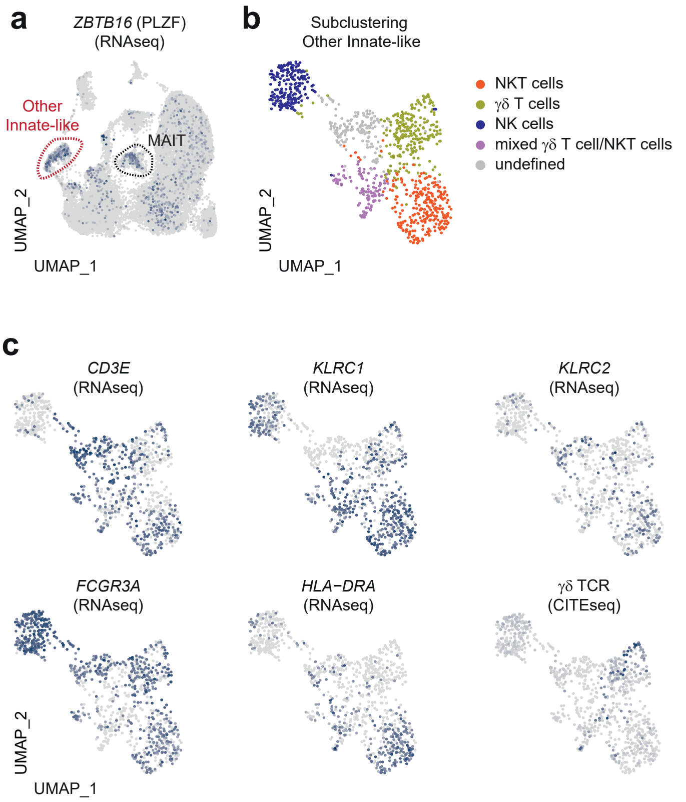


D-G: scRNAseq data set of T cells from kidney biopsies
of ANCA-GN patients (Krebs et al. *Sci Immunol* 2020)[31]

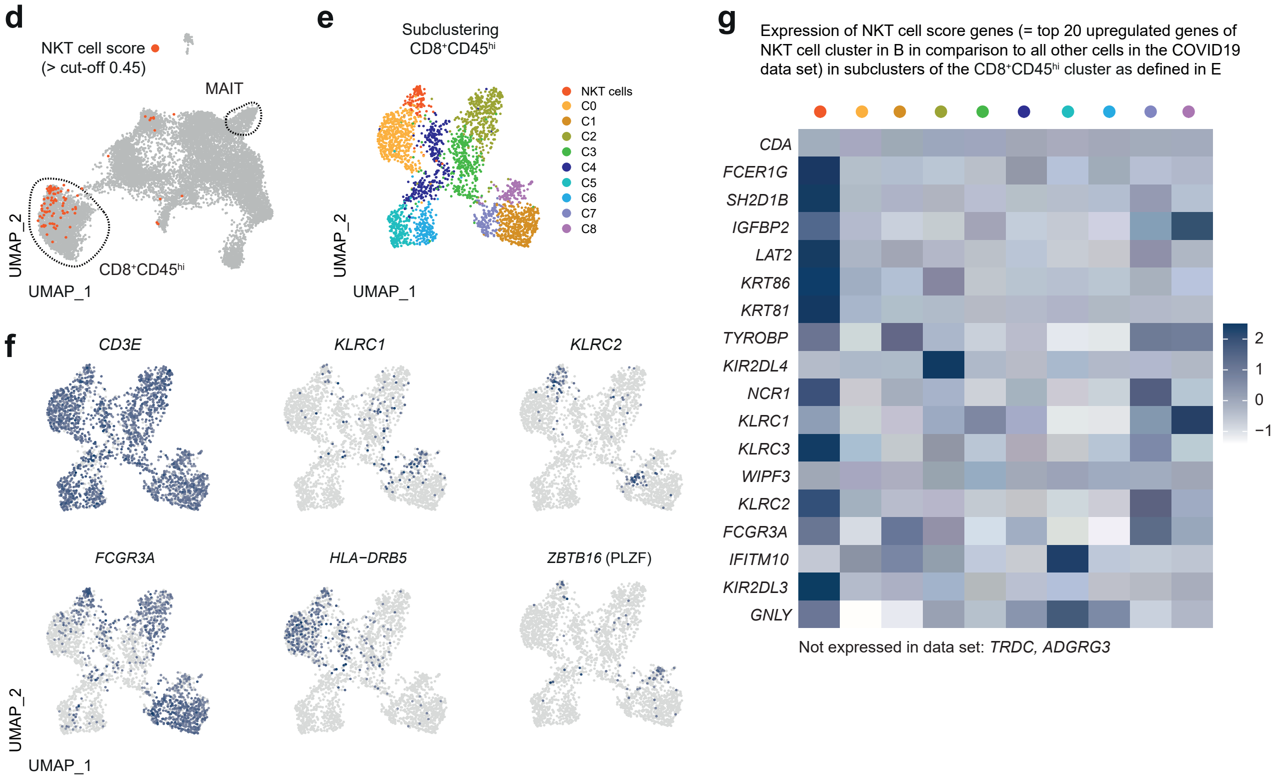

**Figure S2: Identification of Natural Killer T (NKT) cells in single-cell RNA sequencing (scRNAseq) data sets of human T cells. (A-C)** Identification of NKT cells in a published scRNA/CITE-seq (CITE = cellular indexing of transcriptomes and epitopes by sequencing) data set of T cells isolated from the bronchoalveolar lavage fluid (BALF) of Coronavirus Disease 2019 (COVID19) patients[32]. **(A)** Uniform Manifold Approximation and Projection (UMAP) representation of the BALF/COVID19 data set showing expression of *ZBTB16* (encoding for PLZF) as a marker for Mucosal-associated invariant T (MAIT) cells and other innate-like T cells. **(B)** UMAP subclustering of the non-MAIT cell PLZF-expressing "innate-like" cluster. **(C)** Expression of CD3 (mRNA), Natural Killer (NK) / NKT cell markers (mRNA), and the γδ TCR (CITE-seq) in the respective subclusters from B. **(E-G)** Identification of NKT cells in a published scRNAseq data set of T cells isolated from kidney biopsy specimens of anti-neutrophil cytoplasmatic antibody-associated glomerulonephritis (ANCA-GN) patients and unaffected kidney tissue of tumor nephrectomy patients[31]. **(D)** UMAP representation of the ANCA-GN data set. Cells that show high expression of the NKT cell score (= top 20 upregulated genes of the NKT cell cluster in B in comparison to all other cells in the BALF/COVID19 data set) are shown in orange. **(E)** UMAP subclustering of the $CD8^{+}CD45^{hi}$ cluster from D. **(F)** mRNA expression of CD3 and NK / NKT cell markers, and *ZBTB16* in the respective subclusters from E. **(G)** Normalized expression heatmap of the uncurated list of NKT cell score genes (extracted as in D) in the ten clusters of the ANCA-GN data set.

Supplementary Figure 3

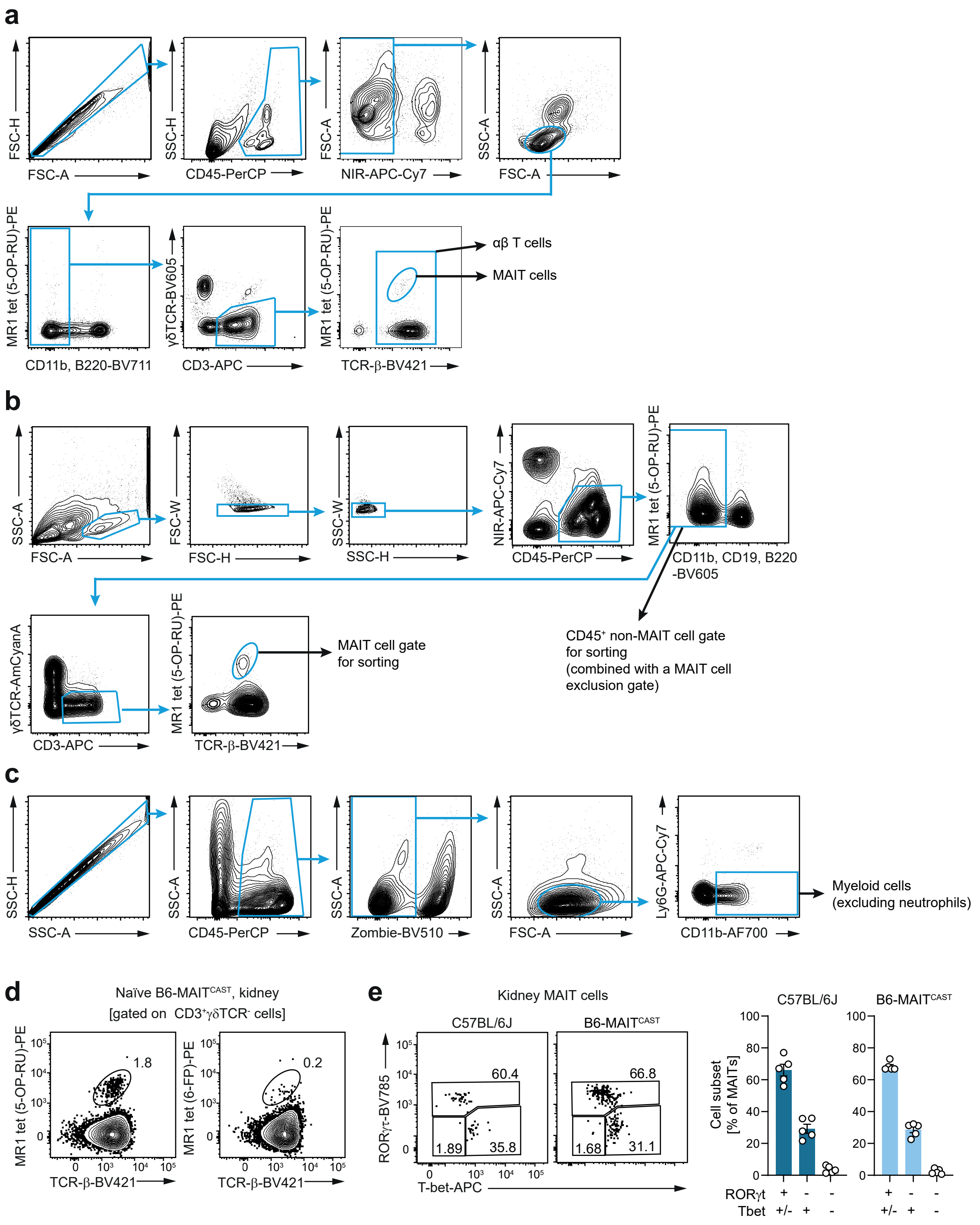


**Figure S3: Gating strategies, specificity of 5-OP-RU-loaded MR1 tetramer staining and transcription factor staining of Mucosal-associated T (MAIT) cells in mice.**

**(A)** Gating strategy for analyses of MAIT cells and αβ T cells in Figures 2, 5, 6, and Supplementary Figures S3, S5-7, S10. **(B)** Gating strategy for sorting of MAIT cells and CD45⁺ non-MAIT cell for single-cell RNA sequencing analyses presented in Figures 3, 5 and Supplementary Figure S4, S8. **(C)** Gating strategy for analyses of Ly6G⁻CD11b⁺ myeloid cells in Figure 5. **(D)** Representative flow cytometry plots of leukocytes isolated from the kidney of from naïve B6-MAIT$^{CAST}$ mice and stained with MR1 tetramers loaded with either the specific MAIT-T cell receptor (TCR) ligand 5-OP-RU or the non-TCR-binding control molecule 6-FP. **(E)** Representative flow cytometry plots of transcription factor staining (left panel) and quantification of MAIT cell subsets (right panel, RORγt$^{+}$T-bet$^{+/-}$ = MAIT17, RORγt$^{-}$T-bet$^{+}$ = MAIT1, RORγt$^{-}$T-bet$^{-}$ = double negative) in kidney MAIT cells from naïve C57BL6/J and B6-MAIT$^{CAST}$ mice (*n* = 5 biologically independent animals per group pooled from two independent experiments). Gating strategy is specified in brackets and numbers indicate the percentage of cells in the gate. Source data are provided as a Source Data file.

Supplementary Figure 4

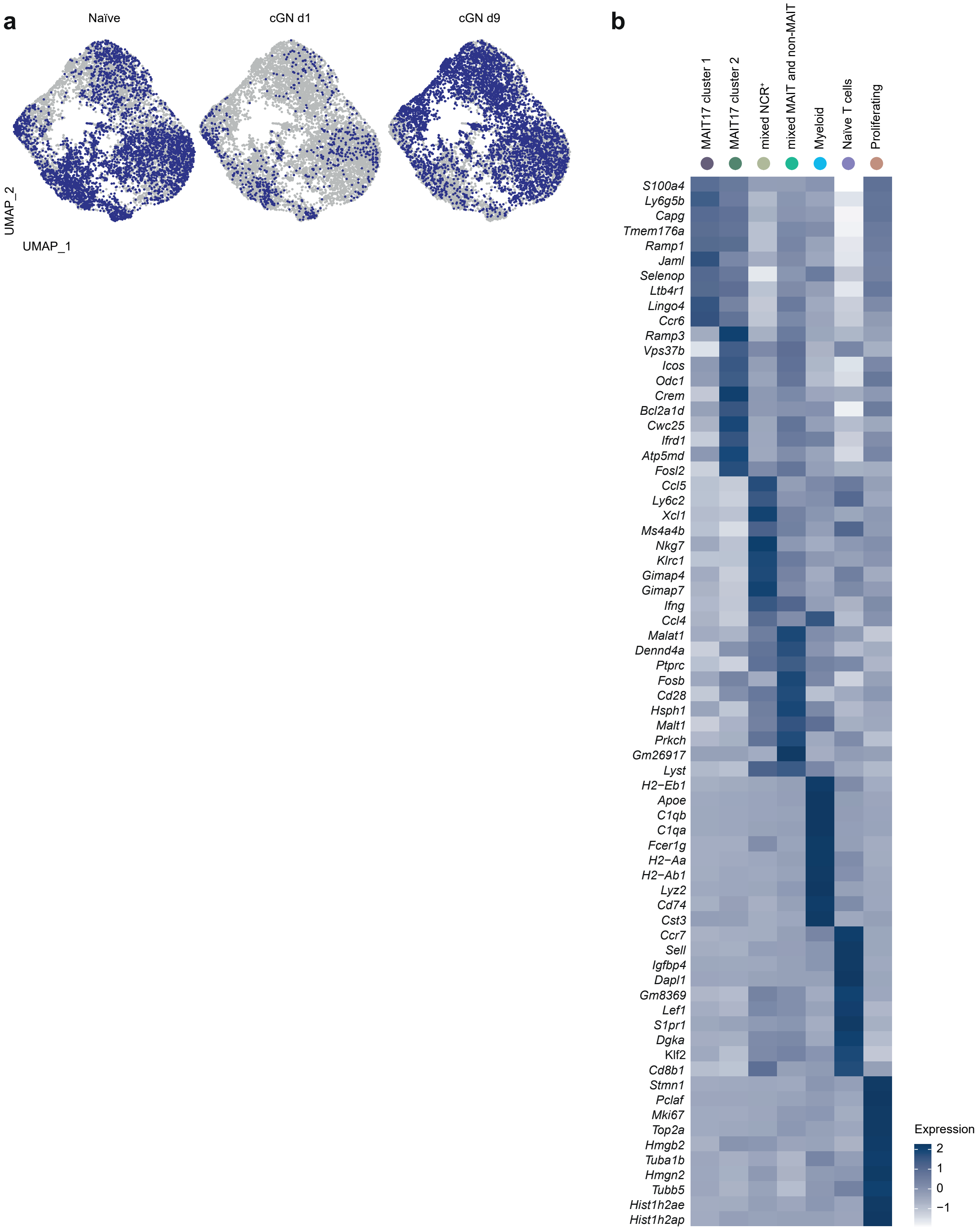


**Figure S4: Cluster distribution and cluster-defining genes in single-cell RNA sequencing (scRNAseq) analysis of kidney Mucosal-associated invariant T (MAIT) cells in experimental crescentic glomerulonephritis (cGN). (A)** Distribution of kidney MAIT cells sorted from B6-MAIT$^{CAST}$ mice (naïve *n* = 18, cGN day 1 *n* = 11, cGN day 9 *n* = 13; *n* represents the number of biologically independent, male, 9-14 week-old mice that were pooled for the isolation) at the different time points on the Uniform Manifold Approximation and Projection plot. **(B)** Normalized expression heatmap of the top ten cluster-defining genes in the seven cell clusters identified by scRNAseq of MR1 tetramer (5-OP-RU)$^{+}$ MAIT cells isolated from the kidney of B6-MAIT$^{CAST}$ mice at the different time points.

Supplementary Figure 5

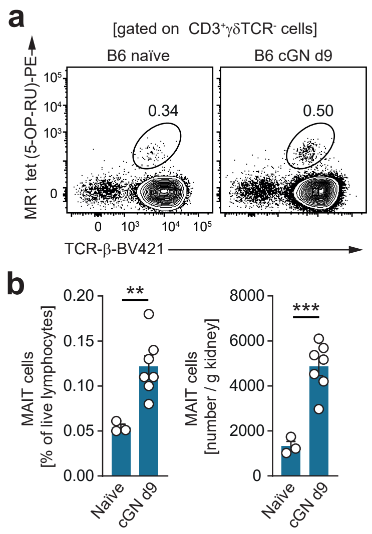


**Figure S5: Accumulation of Mucosal-associated invariant T (MAIT) cells in kidneys of wild type mice with crescentic glomerulonephritis (cGN). (A)** Representative flow cytometry plots of leukocytes isolated from the kidneys of naïve C57BL/6J wild type mice and of C57BL/6J wild type mice on day 9 after induction of cGN. Gating strategy is specified in brackets and numbers indicate the percentage of cells in the gate. **(B)** Frequencies and absolute numbers of MAIT cells in the two groups (B6 naïve $n$ = 3, B6 cGN d9 $n$ = 7; $n$ represents the number biologically independent animals). Data is representative of three independent experiments with similar results. Circles represent individual animals and bars represent mean ± SEM (**$P$ = 0.0085, ***$P$ = 0.0006 in two-sided Student's $t$-Test). Source data are provided as a Source Data file.

Supplementary Figure 6

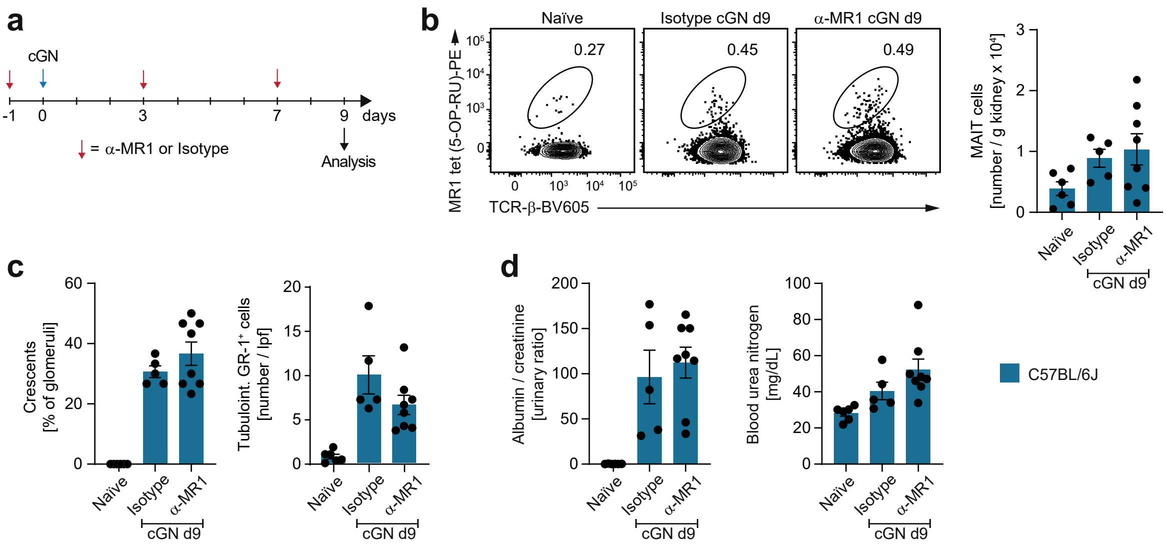


**Figure S6: MR1 blockade does not influence outcome of experimental crescentic glomerulonephritis (cGN) in C57BL/6 wild type mice.**

**(A)** Schematic representation of experimental setup for α-MR1 treatment in cGN. α-MR1 antibody and isotype control were used at a concentration of 200µg per injection (intraperitoneally). **(B)** Representative flow cytometry plots and quantification of Mucosal-associated invariant T cells in kidneys of naïve C57BL/6J wild type mice (*n* = 6 biologically independent animals) and of isotype- (*n* = 5 biologically independent animals) and α-MR1-treated (*n* = 8 biologically independent animals) C57BL/6J wild type with cGN. **(C, D)** Quantification of crescent formation and tubulointerstitial neutrophil infiltration (C) and kidney function parameters (D) in the respective groups. Data are pooled from two independent experiments. Symbols represent individual animals. Bars represent mean ± SEM. Source data are provided as a Source Data file.

Supplementary Figure 7

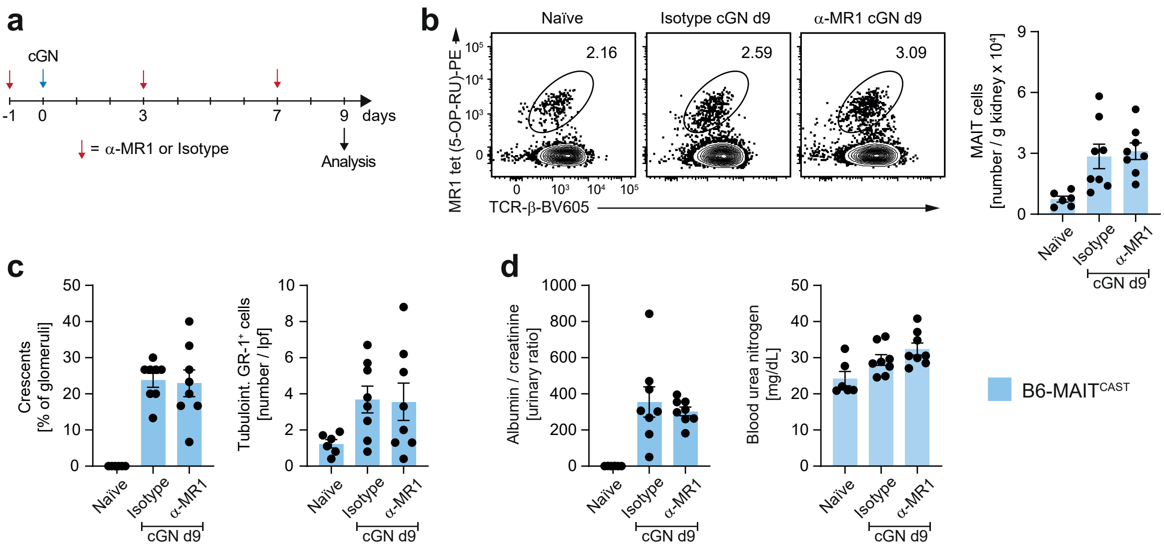


**Figure S7: MR1 blockade does not influence outcome of experimental crescentic glomerulonephritis (cGN) in B6-MAIT$^{CAST}$ mice.**
**(A)** Schematic representation of experimental setup for α-MR1 treatment in cGN. α-MR1 antibody and isotype control were used at a concentration of 200 µg per injection (intraperitoneally). **(B)** Representative flow cytometry plots and quantification of Mucosal-associated invariant T cells in kidneys of naïve B6-MAIT$^{CAST}$ mice (*n* = 6 biologically independent animals) and of isotype- (*n* = 8 biologically independent animals) and α-MR1-treated (*n* = 8 biologically independent animals) B6-MAIT$^{CAST}$ mice with cGN. **(C, D)** Quantification of crescent formation and tubulointerstitial neutrophil infiltration (C) and kidney function parameters (D) in the respective groups. Data are pooled from two independent experiments. Symbols represent individual animals. Bars represent mean ± SEM. Source data are provided as a Source Data file.

Supplementary Figure 8

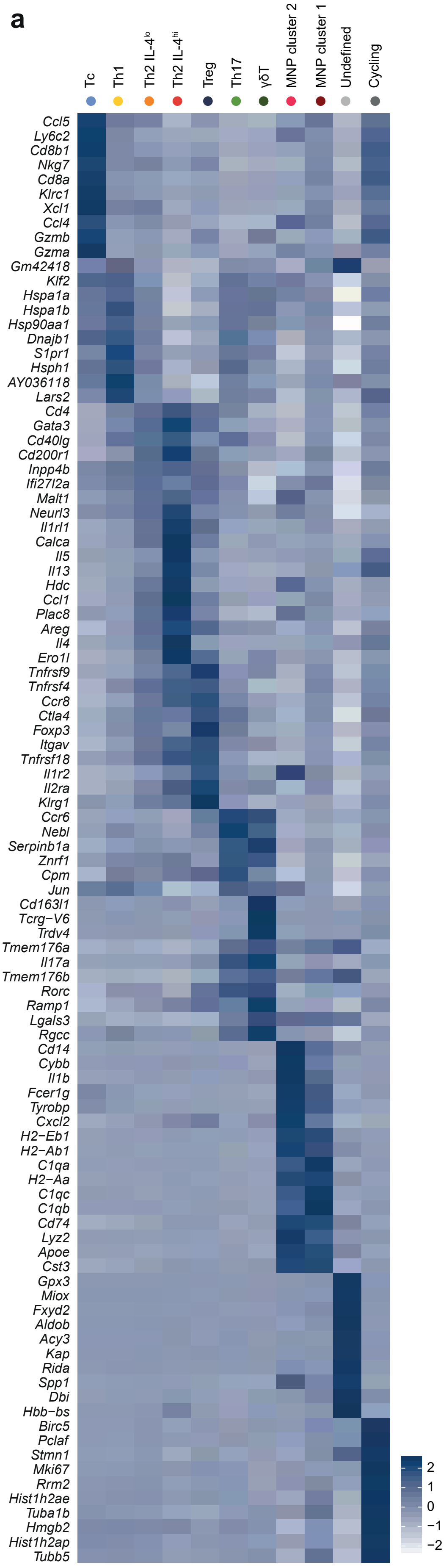


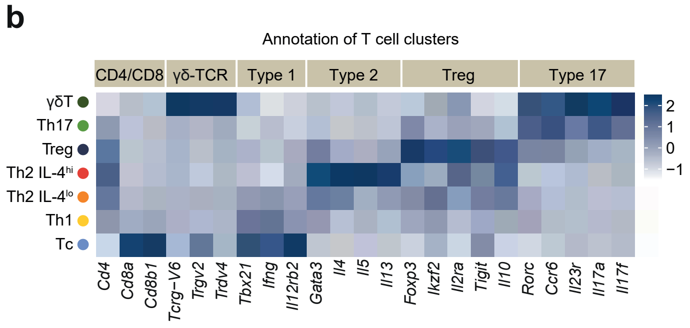


**Figure S8: Expression of cluster-defining genes and T cell subset-defining genes in kidney CD45⁺ non-Mucosal-associated invariant T (non-MAIT) cells in experimental crescentic glomerulonephritis (cGN). (A)** Normalized expression heatmap of the top ten cluster-defining genes in the eleven cell clusters identified by single-cell RNA sequencing of CD45⁺ non-MAIT cells isolated from the kidney of B6-MAIT$^{CAST}$ mice on day 9 after induction of cGN ($n$ = 13; $n$ represents the number of biologically independent, male, 9-14 week-old mice that were pooled for the isolation). **(B)** Normalized expression heatmap of T cell subset-defining genes in the seven T cell clusters in the CD45⁺ non-MAIT cell data set.

Supplementary Figure 9

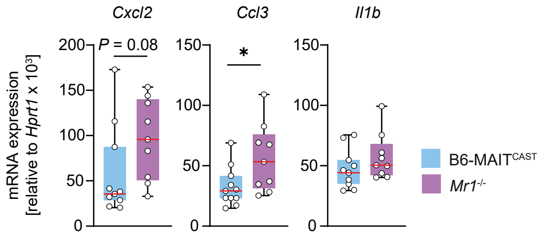


**Figure S9: Absence of Mucosal-associated invariant T (MAIT) cells increases proinflammatory gene expression in the kidney in experimental crescentic glomerulonephritis**. Quantitative RT-PCR analysis of proinflammatory gene expression in the kidney cortex of B6-MAIT$^{CAST}$ and *Mr1*$^{-/-}$ mice on day 9 after induction of cGN (B6-MAIT$^{CAST}$ *n* = 11, *Mr1*$^{-/-}$ *n* = 9). Data are pooled from two independent experiments. Circles represent individual animals and bars represent mean ± SEM. (**P* = 0.0268 in in two-sided Student's *t*-Test). Source data are provided as a Source Data file.

Supplementary Figure 10

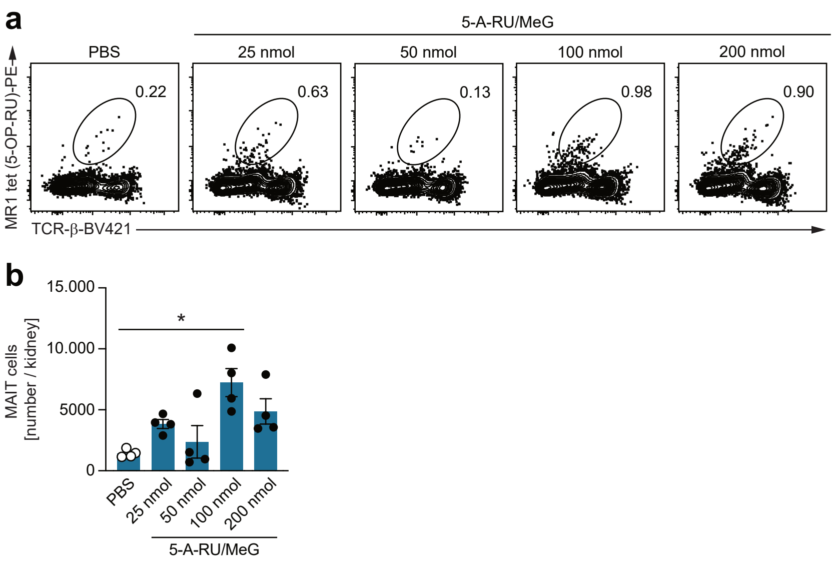


**Figure S10: Expansion of Mucosal-associated invariant T (MAIT) cells in kidneys of mice with crescentic glomerulonephritis (cGN) injected with increasing doses of 5-A-RU/MeG**

**(A)** Representative flow cytometry plots and quantification MAIT cells in kidneys of PBS- and 5-A-RU/MeG-treated C57BL/6J wild type mice on day 10 of cGN. Different doses of 5-A-RU/MeG were injected twice daily starting on day 2 after cGN induction. Concentrations indicated are total daily doses. Numbers indicate percentage of cells in the gate. **(B)** Absolute numbers of MAIT cells in the different groups (*n* = 4 biologically independent animals in each group). Circles represent individual animals and bars represent mean ± SEM (**P* < 0.001 in ordinary one-way ANOVA with Newman-Keuls multiple comparions test). Source data are provided as a Source Data file.
