# Supplementary material for: Mucosal-associated invariant T cells contribute to suppression of inflammatory myeloid cells in immune-mediated kidney disease
Source: Nat Commun. 2023 Nov 15;14:7372. doi: 10.1038/s41467-023-43269-0 (PMC10651937; doi:10.1038/s41467-023-43269-0)
Supplement: Supplementary file 2 — Reporting Summary [file 41467_2023_43269_MOESM2_ESM.pdf]

nature portfolio

Corresponding author(s): Christian Kurts
Jan-Eric Turner

Last updated by author(s): Oct 10, 2023

Reporting Summary

Nature Portfolio wishes to improve the reproducibility of the work that we publish. This form provides structure for consistency and transparency in reporting. For further information on Nature Portfolio policies, see our Editorial Policies and the Editorial Policy Checklist.

Statistics

For all statistical analyses, confirm that the following items are present in the figure legend, table legend, main text, or Methods section.

n/a	Confirmed	
☐	☒	The exact sample size (*n*) for each experimental group/condition, given as a discrete number and unit of measurement
☐	☒	A statement on whether measurements were taken from distinct samples or whether the same sample was measured repeatedly
☐	☒	The statistical test(s) used AND whether they are one- or two-sided<br>*Only common tests should be described solely by name; describe more complex techniques in the Methods section.*
☐	☒	A description of all covariates tested
☐	☒	A description of any assumptions or corrections, such as tests of normality and adjustment for multiple comparisons
☐	☒	A full description of the statistical parameters including central tendency (e.g. means) or other basic estimates (e.g. regression coefficient) AND variation (e.g. standard deviation) or associated estimates of uncertainty (e.g. confidence intervals)
☐	☒	For null hypothesis testing, the test statistic (e.g. *F*, *t*, *r*) with confidence intervals, effect sizes, degrees of freedom and *P* value noted<br>*Give P values as exact values whenever suitable.*
☒	☐	For Bayesian analysis, information on the choice of priors and Markov chain Monte Carlo settings
☒	☐	For hierarchical and complex designs, identification of the appropriate level for tests and full reporting of outcomes
☒	☐	Estimates of effect sizes (e.g. Cohen's *d*, Pearson's *r*), indicating how they were calculated

Our web collection on statistics for biologists contains articles on many of the points above.

Software and code

Policy information about availability of computer code

Data collection	scRNA Sequencing using 10xGenomics Chromium Single Cell 5’v1.1 reagents kit for library preparation. The libraries were sequenced on Illumina NovaSeq 6000 with 150 base pairs and paired-end configurations
Data analysis	Cell Ranger (version 4.0.0) was used to demultiplex cellular barcodes and map reads to the reference genome (mm10-Ensemble 98)<br>Data analysis was performed using R (Version 4.0.3).<br>For clustering: FindIntegrationAnchors function and IntegrateData function of Seurat (v.4.0.0)<br>UMAP: using Seurats FindClusters function with a resolution of 0.2 for MAIT cells and 0.5 for non-MAIT cells.<br>murine MAIT cell activation score: AddModuleScore function with default parameters.<br>Differential expression analyses: using FindAllMarkers function of Seurat.<br>Interactome analysis: using CellPhoneDB (Version 2.1.4) and layout.fruchterman.reingold function of the R package iGraph in combination with the plot function<br><br>Analysis of published scRNAseq dataset.<br>Human MAIT cell-defining genes: using the FindMarkers function of the R package Seurat (v.4.0.0).<br>human MAIT cell activation score: using AddModuleScore function with default parameters of the R package Seurat.

For manuscripts utilizing custom algorithms or software that are central to the research but not yet described in published literature, software must be made available to editors and reviewers. We strongly encourage code deposition in a community repository (e.g. GitHub). See the Nature Portfolio guidelines for submitting code & software for further information.

Data

Policy information about availability of data

All manuscripts must include a data availability statement. This statement should provide the following information, where applicable:

- Accession codes, unique identifiers, or web links for publicly available datasets
- A description of any restrictions on data availability
- For clinical datasets or third party data, please ensure that the statement adheres to our policy

GEO Series accession number GSE245302 (https://www.ncbi.nlm.nih.gov/geo/query/acc.cgi?acc=GSE245302)

Research involving human participants, their data, or biological material

Policy information about studies with human participants or human data. See also policy information about sex, gender (identity/presentation), and sexual orientation and race, ethnicity and racism.

Reporting on sex and gender	This study involved the reanalysis of a human data set previously published by our group (Sci Immunol. 2020 Aug 7;5(50):eaba4163).<br>In this study kidney biopsies of 13 patients with ANCA-GN (2 female, 11 male patients) and 6 control biopsies after tumor nephrectomy (3 female, 3 male patients) were analyzed. Please refer to the original publication for further information.
Reporting on race, ethnicity, or other socially relevant groupings	Patients with ANCA-GN were at the age of 19-82 years at the time of biopsy. Patients after tumor nephrectomy were at the median age of 49,9 (+/- 16.5) years. For detailed information please see the published data set. (Sci Immunol. 2020 Aug 7;5(50):eaba4163)
Population characteristics	For the published study, human tissue samples were obtained from the healthy part of kidneys after tumor nephrectomy. For some of the analyses, matched blood samples from the same patient were used. Samples were provided in an anonymized fashion, precluding the analysis of population characeristics.
Recruitment	All patients were recruited at the University Medical Center Hamburg-Eppendorf. Informed consent was obtained from all participating patients.
Ethics oversight	These studies were approved by the Ethik-Kommission der Ärztekammer Hamburg (local ethics committee of the chamber of physicians in Hamburg) und the registration number PV 5822, and were conducted in accordance with the ethical principles stated by the Declaration of Helsinki.

Note that full information on the approval of the study protocol must also be provided in the manuscript.

Field-specific reporting

Please select the one below that is the best fit for your research. If you are not sure, read the appropriate sections before making your selection.

☒ Life sciences ☐ Behavioural & social sciences ☐ Ecological, evolutionary & environmental sciences

For a reference copy of the document with all sections, see nature.com/documents/nr-reporting-summary-flat.pdf

Life sciences study design

All studies must disclose on these points even when the disclosure is negative.

Sample size	Sample size was determined based on previous experience with the models and complexity of the experiments. No statistical method was used to predetermine sample size. Animals were randomly assigned to different treatment groups.
Data exclusions	No data were excluded from the analyses of animal outcome experiments. For scRNAseq data: Exclusion of low quality cells (<200 genes expressed), low expressed genes (expressed in less than 3 cells) and cells with upregulated mitochondrial genes (more than 5 % of all reads).
Replication	All animal experiments were performed at least twice with similar results. The number of replications for each experiment is indicated in the Figure legend.
Randomization	or cell isolation, samples have been prepared independent of their group (not group-wise).<br>Human samples were analyzed by comparing disease conditions with healthy controls in an observational manner.
Blinding	The Investigators were not blinded to allocation during experiments, but all histology samples for assessment of cGN outcome were analyzed in a blinded fashion.

Reporting for specific materials, systems and methods

We require information from authors about some types of materials, experimental systems and methods used in many studies. Here, indicate whether each material, system or method listed is relevant to your study. If you are not sure if a list item applies to your research, read the appropriate section before selecting a response.

Materials & experimental systems

n/a	Involved in the study
☐	☒ Antibodies
☒	☐ Eukaryotic cell lines
☒	☐ Palaeontology and archaeology
☐	☒ Animals and other organisms
☐	☐ Clinical data
☒	☐ Dual use research of concern
☒	☐ Plants

Methods

n/a	Involved in the study
☒	☐ ChIP-seq
☐	☒ Flow cytometry
☒	☐ MRI-based neuroimaging

Antibodies

Antibodies used

Human:
CD45 (HI30, Biolegend, https://www.biolegend.com/en-us/products/purified-anti-human-cd45-antibody-710?GroupID=BLG5926)
CD3e (OKT3, Biolegend, https://www.biolegend.com/en-us/products/purified-anti-human-cd3-antibody-3642)
gdTCR (B1, Biolegend, https://www.biolegend.com/en-us/products/purified-anti-human-tcr-gamma-delta-antibody-4546?GroupID=BLG5697)
CD161 (HP-3G10, Biolegend, https://www.biolegend.com/en-us/products/purified-anti-human-cd161-antibody-5672?GroupID=BLG9768)
CD127 (A019D5, Biolegend, https://www.biolegend.com/nl-nl/products/purified-anti-human-cd127-il-7ralpha-antibody-7093)
CD69 (FN50, Biolegend, https://www.biolegend.com/en-us/products/purified-anti-human-cd69-antibody-1670)
IL-18R (H44, Biolegend, https://www.biolegend.com/fr-ch/products/purified-anti-human-cd218a-il-18ralpha-antibody-2616?GroupID=BLG10057)
RORgt (Q21-559, BD Biosciences, https://www.bdbiosciences.com/en-br/products/reagents/flow-cytometry-reagents/research-reagents/single-color-antibodies-ruo/pe-mouse-anti-human-ror-t.563081)

Mr1-Tetramer (5-OP-RU) (39237, 2018, NIH, https://tetramer.yerkes.emory.edu/reagents/mr1)
Mr1-Tetramer (6-FP) (39238, 2018, NIH, https://tetramer.yerkes.emory.edu/reagents/mr1)

Mouse:
CD45 (30-F11, BioLegend, https://www.biolegend.com/en-us/products/purified-anti-mouse-cd45-antibody-102?GroupID=BLG1932)
CD3 (145-2C11, BD Biosciences, https://www.bdbiosciences.com/en-in/products/reagents/flow-cytometry-reagents/research-reagents/single-color-antibodies-ruo/purified-hamster-anti-mouse-cd3e.557306)
CD4 (RM4-5, BioLegend, https://www.biolegend.com/nl-nl/products/purified-anti-mouse-cd4-antibody-484)
CD8 (53-6.7, BioLegend, https://www.biolegend.com/nl-be/products/purified-anti-mouse-cd8a-antibody-157)
TCR-β (H57-597, BioLegend, https://www.biolegend.com/de-de/products/purified-anti-mouse-tcr-beta-chain-antibody-274)
TCR-γδ (GL3, BD Biosciences and BioLegend, https://www.biolegend.com/fr-lu/products/purified-anti-mouse-tcr-gamma-delta-antibody-2418?Clone=GL3)
CD11b (M1/70, BioLegend, https://www.biolegend.com/en-us/products/purified-anti-mouse-human-cd11b-antibody-351?GroupID=BLG10660)
Ly6G (1A8, BioLegend, https://www.biolegend.com/en-us/products/purified-anti-mouse-ly-6g-antibody-4767?GroupID=BLG7232)
B220 (RA3-6B2, BioLegend, https://www.biolegend.com/en-gb/cell-health/purified-anti-mouse-human-cd45r-b220-antibody-449?GroupID=GROUP658)
CD69 (H1.2F3, BioLegend, https://www.biolegend.com/en-us/products/purified-anti-mouse-cd69-maxpar-ready-antibody-10091)
CD127 (A7R34, BioLegend, https://www.biolegend.com/en-gb/products/purified-anti-mouse-cd127-il-7ralpha-antibody-6188)
CD103 (2E7, BioLegend, https://www.biolegend.com/nl-be/products/purified-anti-mouse-cd103-antibody-3572)
CD44 (IM7, BioLegend, https://www.biolegend.com/en-us/products/purified-anti-mouse-human-cd44-antibody-318?GroupID=BLG10425)
CXCR6 (SA051D1, BioLegend, https://www.biolegend.com/de-de/products/purified-anti-mouse-cd186-cxcr6-antibody-12544?GroupID=BLG14919)
CD25 (QA19A49, Biolegend, https://www.biolegend.com/nl-be/products/purified-anti-mouse-cd25-recombinant-antibody-20645?GroupID=ImportedGROUP1)
IL18R (REA947, Miltenyi, https://www.miltenyibiotec.com/DE-en/products/cd218a-il-18ra-antibody-anti-mouse-reafinity-rea947.html#conjugate=fitc:size=30-ug-in-200-ul; BG/IL18RA, BioLegend)
IL-1β (NJTEN3, Invitrogen, https://www.thermofisher.com/antibody/product/IL-1-beta-Pro-form-Antibody-clone-NJTEN3-Monoclonal/25-7114-82)
CCL3 (DNT3CC, Invitrogen, https://www.thermofisher.com/antibody/product/CCL3-MIP-1-alpha-Antibody-clone-DNT3CC-Monoclonal/50-7532-82)
CD152 (UC10-4B9, BioLegend, https://www.biolegend.com/de-at/products/purified-anti-mouse-cd152-antibody-517?GroupID=BLG10448)
CXCL16 (12-81, BD Biosciences,https://www.bdbiosciences.com/en-us/products/reagents/flow-cytometry-reagents/research-reagents/single-color-antibodies-ruo/pe-rat-anti-mouse-cxcl16.566740)
IL-17A (TC11-18H10.1, BioLegend, https://www.biolegend.com/fr-lu/products/purified-anti-mouse-il-17a-antibody-1634?GroupID=GROUP24)
IFN-γ (XMG1.2, BioLegend, https://www.biolegend.com/de-de/cell-health/purified-anti-mouse-ifn-gamma-antibody-998)
IL-4 (11B11, BioLegend, https://www.biolegend.com/en-gb/products/purified-anti-mouse-il-4-antibody-894)
GATA-3 (L50-823, BD Biosciences, https://www.bdbiosciences.com/en-us/products/reagents/microscopy-imaging-reagents/

	immunofluorescence-reagents/purified-mouse-anti-gata3.558686)<br>T-bet (4B10, BioLegend, https://www.biolegend.com/fr-fr/products/purified-anti-t-bet-antibody-5757)<br>RORγt (Q31-378, BD Biosciences, https://www.bdbiosciences.com/en-au/products/reagents/flow-cytometry-reagents/research-reagents/single-color-antibodies-ruo/purified-mouse-anti-mouse-ror-t.562663)<br>Mr1-Tetramer (5-OP-RU) (50922, 2020, NIH, https://tetramer.yerkes.emory.edu/reagents/mr1)<br>Mr1-Tetramer (6-FP) (50921, 2020, NIH, https://tetramer.yerkes.emory.edu/reagents/mr1)<br><br>Neutralization antibodies:<br>anti-IgG2a (MAB003, R&D, https://www.rndsystems.com/products/mouse-igg-2a-isotype-control_mab003)<br>anti-CXCL16 (MAB503, R&D, https://www.rndsystems.com/products/mouse-cxcl16-antibody-142417_mab503?gclid=Cj0KCQjwj5mpBhDJARIsAOVjBdreUxL1YQntSVGRwSTUPzjdPfg8s0etHUfo0iGZ_CnUdNE1AKWXddoaAq21EALw_wcB&gclsrc=aw.ds)
Validation	Human antibodies have been tested and validated by flowcytometric analyses using peripheral mononuclear leukocytes. Mouse antibodies have been tested and validated by flowcytometric analyses using cells isolated from spleen and kidney.<br>For detailed information regarding the technical data sheets, manufacter's validation statements and relevant citations please see the links above. Please see Methods Section for the antibody dilutions used in the experiments.

Animals and other research organisms

Policy information about studies involving animals; ARRIVE guidelines recommended for reporting animal research, and Sex and Gender in Research

Laboratory animals	B6.129P2-Mr1tm1Gfn (Mr1-/-) and B6(Cg)-MaithiRorctm2Litt (B6-MAITCAST), C57BL6/J
Wild animals	The study did not involve wild animals.
Reporting on sex	In all experiments adult (> 8 week-old), sex- and age-matched mice were used. For further information please see Methods section and the source data file.
Field-collected samples	The study did not apply field-collected samples.
Ethics oversight	All animal experiments were performed according to national and institutional animal care and ethical guidelines and were approved by the Behörde für Gesundheit und Verbraucherschutz, Freie und Hansestadt Hamburg (local committees of the city of Hamburg, Germany) under the approval number N020/2022.

Note that full information on the approval of the study protocol must also be provided in the manuscript.

Clinical data

Policy information about clinical studies

All manuscripts should comply with the ICMJE guidelines for publication of clinical research and a completed CONSORT checklist must be included with all submissions.

Clinical trial registration	*Provide the trial registration number from ClinicalTrials.gov or an equivalent agency.*
Study protocol	*Note where the full trial protocol can be accessed OR if not available, explain why.*
Data collection	*Describe the settings and locales of data collection, noting the time periods of recruitment and data collection.*
Outcomes	*Describe how you pre-defined primary and secondary outcome measures and how you assessed these measures.*

Plants

Seed stocks	*Report on the source of all seed stocks or other plant material used. If applicable, state the seed stock centre and catalogue number. If plant specimens were collected from the field, describe the collection location, date and sampling procedures.*
Novel plant genotypes	*Describe the methods by which all novel plant genotypes were produced. This includes those generated by transgenic approaches, gene editing, chemical/radiation-based mutagenesis and hybridization. For transgenic lines, describe the transformation method, the number of independent lines analyzed and the generation upon which experiments were performed. For gene-edited lines, describe the editor used, the endogenous sequence targeted for editing, the targeting guide RNA sequence (if applicable) and how the editor was applied.*
Authentication	*Describe any authentication procedures for each seed stock used or novel genotype generated. Describe any experiments used to assess the effect of a mutation and, where applicable, how potential secondary effects (e.g. second site T-DNA insertions, mosiacism, off-target gene editing) were examined.*

Flow Cytometry

Plots

Confirm that:

☒ The axis labels state the marker and fluorochrome used (e.g. CD4-FITC).

☒ The axis scales are clearly visible. Include numbers along axes only for bottom left plot of group (a 'group' is an analysis of identical markers).

☒ All plots are contour plots with outliers or pseudocolor plots.

☒ A numerical value for number of cells or percentage (with statistics) is provided.

Methodology

Sample preparation	Cell isolation from human tissue sample and mice organs were prepared as described in the Methods Section. Samples were stained according to the manufacturer's recommendations.
Instrument	Flow Cytometry Analysis: FACS LSR II. Cell sorting: FACS AriaFusion. All BD Biosciences
Software	BD FACS Diva version 8.0.1. Analysis: FlowJo version 10.8.1
Cell population abundance	After sorting, we typically had a purity of 95-99% of sorted MAIT cells.
Gating strategy	MAIT cell sort gating strategy: Lymphocytes (FSC/SSC), CD45+, Live (NIR-negative), B220-CD19-, gdTCR-CD3+, TCRb+Tetramer (5-OP-RU)+. Please see Figure S3 for further information on gating stragegies.

☒ Tick this box to confirm that a figure exemplifying the gating strategy is provided in the Supplementary Information.
